# Clinical responses to ERK inhibition in *BRAF*^V600E^-mutant colorectal cancer predicted using a computational model

**DOI:** 10.1038/s41540-017-0016-1

**Published:** 2017-06-02

**Authors:** Daniel C. Kirouac, Gabriele Schaefer, Jocelyn Chan, Mark Merchant, Christine Orr, Shih-Min A. Huang, John Moffat, Lichuan Liu, Kapil Gadkar, Saroja Ramanujan

**Affiliations:** Genentech Research & Early Development, 1 DNA Way, South San Francisco, CA 94080 USA

## Abstract

Approximately 10% of colorectal cancers harbor *BRAF* ^V600E^ mutations, which constitutively activate the MAPK signaling pathway. We sought to determine whether ERK inhibitor (GDC-0994)-containing regimens may be of clinical benefit to these patients based on data from in vitro (cell line) and in vivo (cell- and patient-derived xenograft) studies of cetuximab (EGFR), vemurafenib (BRAF), cobimetinib (MEK), and GDC-0994 (ERK) combinations. Preclinical data was used to develop a mechanism-based computational model linking cell surface receptor (EGFR) activation, the MAPK signaling pathway, and tumor growth. Clinical predictions of anti-tumor activity were enabled by the use of tumor response data from three Phase 1 clinical trials testing combinations of EGFR, BRAF, and MEK inhibitors. Simulated responses to GDC-0994 monotherapy (overall response rate = 17%) accurately predicted results from a Phase 1 clinical trial regarding the number of responding patients (2/18) and the distribution of tumor size changes (“waterfall plot”). Prospective simulations were then used to evaluate potential drug combinations and predictive biomarkers for increasing responsiveness to MEK/ERK inhibitors in these patients.

## Systems pharmacology:Predicting efficacy of novel anti-cancer drugs in colorectal cancer

While cancer drug development relies on experimental tumor models for testing, results observed in these systems often fail to translate clinically. Kirouac et al. demonstrate how computational systems modelling can help bridge this divide. Focusing on a class of colorectal cancers with poor prognosis (those with a mutant form of the *BRAF* oncogene) they develop a mathematical model linking drug exposure, via cellular signal transduction, to tumor growth. By triangulating experimental data from multiple cell lines and mouse models, with results from three clinical trials of related drugs, the model accurately predicted tumor shrinkage observed in a first-in-human study of GDC-0994, an ERK inhibitor. Simulations were then used to explore strategies for increasing the activity of this class of drugs (MAPK pathway inhibitors) via combinations, alternate dosing regimens, and predictive biomarkers to guide future clinical studies. Extended to other cancer types and drugs, the approach could streamline early clinical development.

## Introduction

Approximately 50% of melanomas and 10% of colorectal cancers (CRC) harbor V600E/K point mutations in the cytosolic kinase *BRAF*. While receptor-mediated activation of RAS-GTP normally regulates activity of the enzyme by catalyzing the formation of BRAF dimers, V600 mutations result in constitutive signaling by the *BRAF* monomer, and subsequent MEK and ERK phosphorylation.^1^ The effector kinase ERK phosphorylates over 100 cytosolic and nuclear substrates, which regulate enzymatic activity and gene expression, promoting cell proliferation and survival.^2^

Vemurafenib (Zelboraf®) and dabrafenib (Tafinlar®) are ATP-competitive BRAF inhibitors (BRAFi) highly selective for the V600E-mutant, both approved for the treatment of metastatic melanoma.^3–5^ While approximately half of melanoma patients harboring this mutation respond to single agent therapy, the duration of response is typically less than a year. Initial responses correlate with the degree of phospho-ERK (pERK) suppression^6^ and resistance is often associated with reactivation of MAPK/ERK signaling via a multitude of genetic and epigenetic mechanisms.^7–10^ Combination with the MEK inhibitors (MEKi) cobimetinib (Cotellic®) or trametinib (Mekinst®) achieves more robust pERK suppression, increasing overall response rates (ORR) to greater than 70%, and extending both progression free survival and overall survival.^11–13^

Following impressive responses in melanoma, BRAFi, and MEKi therapies have been tested in *BRAF* ^V600E^ mutant cancers of other origins such as colorectal.^14^ The clinical activity observed in small initial trials has, however, been quite modest in comparison to melanoma patients, with response rates of 5% for vemurafenib monotherapy,^15^ and 12% for BRAFi + MEKi (dabrafenib and trametenib) combinations.^16^ Given the high expression and activity of epidermal growth factor receptor (EGFR) in CRC, “by-pass” signaling through EGFR/RAS/CRAF was postulated to mediate BRAFi resistance in pre-clinical models.^17, 18^ The addition of an EGFR blocking antibodies (EGFRi) such as cetuximab or panitumumab to BRAFi and MEKi regimens has yielded modest additional clinical benefit (ORR = 26% to the triple combination),^19^ but still far less than achievable in melanoma. As such, there remains an unmet medical need to find more effective therapies for the treatment of *BRAF* ^V600E^-CRC.

If such cancers are dependent upon MAPK signaling, we reasoned that ERK inhibitors (ERKi) either alone or as part of multi-drug regimens could be of clinical benefit in *BRAF* ^V600E^-CRC, given the pre-clinical activity observed in melanoma and CRC models resistant to BRAF and MEK inhibitors.^20–23^ A number of ERK inhibitors, including BVD-523, SCH772984, and GDC-0994 (ref. 24) are currently in pre-clinical and early clinical development, but it remains unclear if these agents will show more favorable clinical activity compared to MEK inhibitors.

For the predictive evaluation of such novel anticancer agents, patient-derived xenografts (PDX) are emerging as valuable tool.^25^ However, systematic testing of alternate dosing regimens and combinations in panels of genetically diverse, clinically representative tumor models via “PDX clinical trials”^26^ is tedious and impractical as a drug development platform. As an alternative, we developed a predictive computational model of the MAPK pathway and its regulation of tumor growth in *BRAF* ^V600E^-CRC, using a quantitative systems pharmacology-based approach,^27^ which:

I. Quantitatively reproduces in vitro (cell line) signaling responses and genetic mechanisms of resistance to targeted (MAPK pathway) inhibitors
II. Predicts in vivo (cell line and patient-derived xenograft) tumor growth responses to drug treatments based on these underlying biochemical and cellular mechanisms
III. Reproduces available clinical data on tumor size changes in response to EGFRi, BRAFi, and MEKi treatments
IV. Predicts clinical responses to a novel ERKi (GDC-0994)^28^ monotherapy and combination regimens

## Results

### A mathematical model of the MAPK pathway including multiple signaling feedbacks and redundanceis

**Fig. 1:**
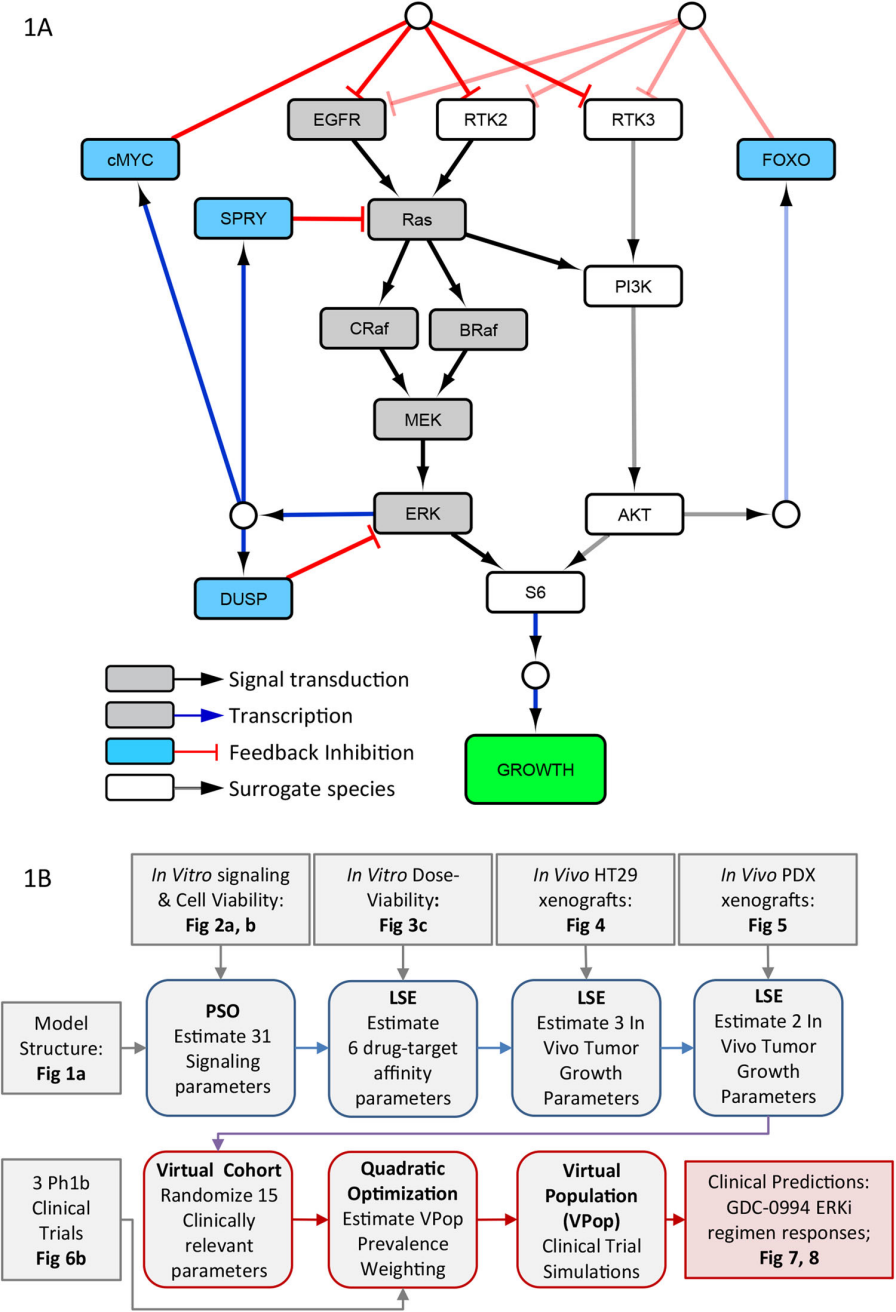
MAPK signaling model structure and development workflow. **a** Model structure. *Gray nodes* indicate core MAPK signal signaling components, *blue nodes* represent regulatory feedback components, and *white nodes* surrogate alternate pathway (PI3K/Akt), alternate receptors (RTK2, 3), and signal integration (S6) components. **b** Model development workflow, highlighting data inputs (*gray boxes*), pre-clinical modeling tasks (*blue boxes*), and clinical translation modeling tasks (*red boxes*), and associated figures. *PSO* particle swarm optimization, LSE least squares estimation

Based on current scientific understanding and public literature, we constructed a mathematical model representing the MAPK/ERK signaling pathway (Fig. 1a). The model is focused around the canonical cascade connecting EGFR, RAS, RAF (BRAF and CRAF), MEK, and ERK. Alternate receptors such as MET, FGFR, and PDGFR are also known to activate the MAPK cascade, and these were lumped together as *RTK2*. Three negative feedback circuits initiated by kinase active ERK were included. DUSP phosphatases which inhibit ERK directly, Sprouty (SPRY) which blocks RAS activation,^7^ and transcriptional circuits mediated by *MYC*, which inhibit the expression and activity of EGFR and other RTKs.^29^ Many additional feedback mechanisms have been described in the literature, such as inhibitory phosphorylation of MEK, CRAF, and EGF by ERK.^7^ However, the functional effects of such additional mechanisms would not be discernable from the three already considered given the data, and were thus omitted.

The PI3K/AKT cascade functionally compensates for MAPK/ERK signaling in certain contexts,^30, 31^ and thus was also represented in the model. Various receptors are known to signal through PI3K, such as ERBB-family members and IGF1R. Receptors that can drive PI3K activation but only weakly influence MAPK are represented as *RTK3*. PI3K signaling can also be activated by the same receptors that drive MAPK activation (*EGFR* and *RTK2*), either directly or through RAS.^1^ Since the exact mechanism of PI3K activation cannot be differentiated from the data used, RAS-mediated activation is used to represent the net effect of these two mechanisms. The two cascade outputs, ERK and AKT, are then integrated by the cell’s translational and transcriptional machinery. Other oncogenic pathways such as Wnt/β-catenin, JNK/c-Jun, or Notch signaling may also functionally compensate for MAPK/ERK-driven tumor growth. As such, the PI3K/AKT branch represented in the model serves as a surrogate for alternate (non-MAPK) oncogenic pathways. Similarly, while signal integration is represented in the model diagram by a single protein S6, this serves as a surrogate for a multi-faceted process including but not limited to metabolic regulation via mTOR, protein translation through 4E-BP, and transcriptional regulation via AP-1 (ref. 1). An empirical time delay term connects cytosolic signaling flux, through dynamic changes in gene expression and protein activity, to cell growth. The final model structure comprises 38 species and 103 parameters, implemented as a system of differential-algebraic equations (Tables S1, S2, and Methods). Our model development workflow is summarized in Fig. 1b, successively incorporating in vitro cell signaling and cell viability, in vivo xenograft tumor growth kinetics, and finally tumor size changes from Phase 1b clinical trials, toward predicting the clinical activity of novel drug combination treatments including the ERKi GDC-0994.

### Model simulations recapitulate in vitro signaling dynamics, cell growth responses, and genetic mechanisms of resistance to drug treatments

We first assessed whether the model could be calibrated to recapitulate published data on cell signaling and growth responses to MAPK inhibitors in *BRAF*-mutant cell lines. Two key results were considered. First, in *BRAF*-mutant melanoma cell lines BRAFi treatment results in robust suppression of pERK, whereas in *BRAF*-mutant CRC cell lines, BRAFi treatment leads to transient pERK suppression for upto 24 h, followed by a rebound in its activity to approximately 40% of the baseline by 48 h (ref. 18). The pERK “rebound” has been attributed, via gene knockout and inhibitor studies, to the activation of EGFR, which is expressed at much higher levels in CRC than in melanoma.^17^

**Fig. 2:**
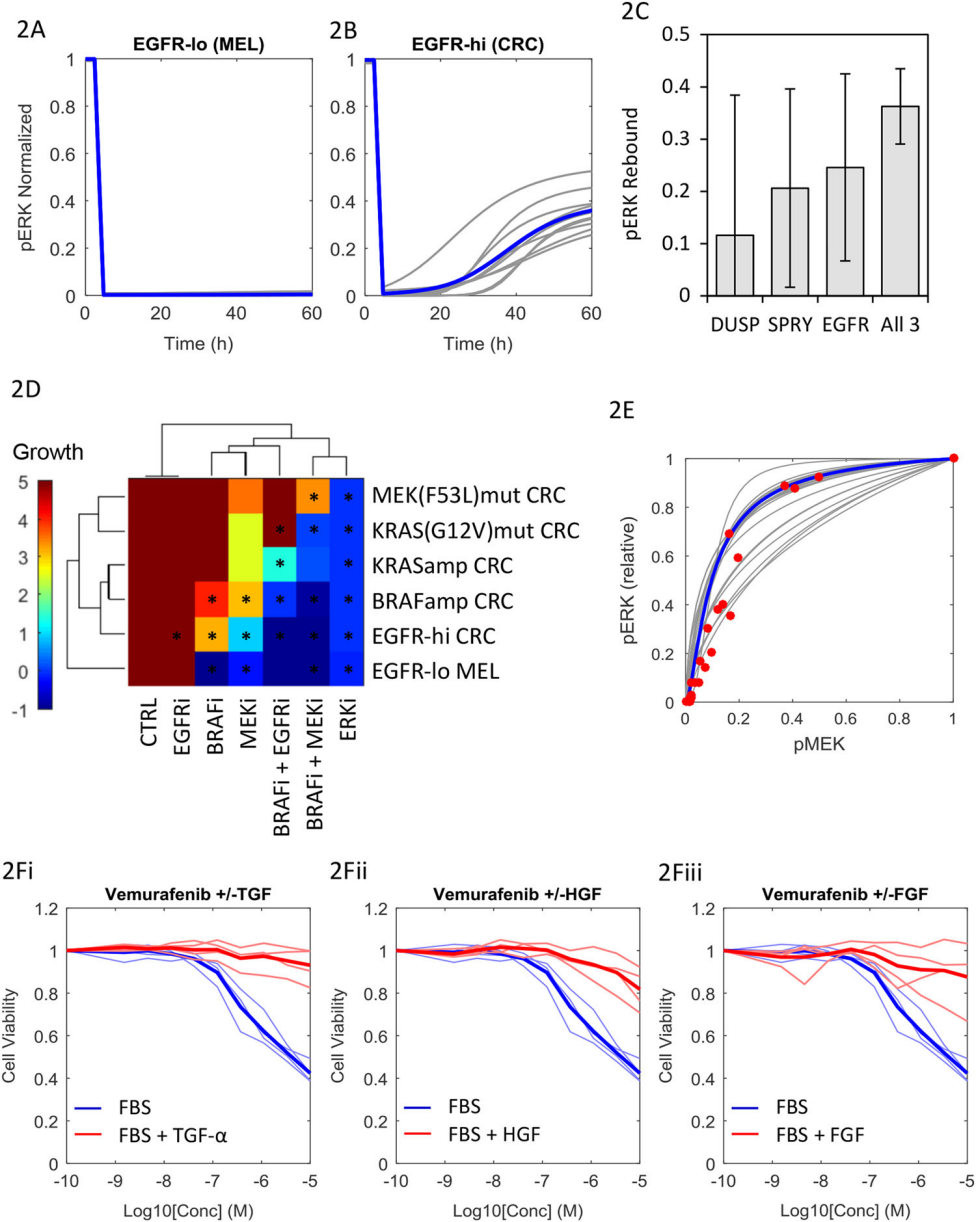
The MAPK model reproduces published in vitro signaling and drug sensitivity data. pERK dynamics in response to BRAFi treatment in EGFR^lo^ melanoma cells **a** and EGFR^hi^ CRC cells **b**. **c** Degree of pERK ‘‘rebound’’ with the three potential feedback mechanisms switched on in isolation, and simultaneously, error bars indicating std across parameter sets. **d** Simulated cell growth (fold expansion) over 72 h for six variant cell lines with six drug treatments. *Asterisks* indicate conditions with matching data.^18, 23, 32^ **e** Relationship between steady-state pMEK and pERK. *Gray lines* are simulations of 20 alternate model parameter sets; *blue line* is a simulation of the Schoeberl (2002) mechanism-based biochemical model,^34^ and *red dots* are quantitative western blot data from four *BRAF*-CRC cell lines treated with the pan-RAFi AZ628 (ref. 32). **f** Viability of four *BRAF*-CRC cell lines cultured in the presence (*red*) or absence (*blue*) of TGF-α, HGF, or FGF ligands. *Thick lines* indicate median responses

We used a particle swarm optimization (PSO) algorithm to search for a set of 28 system parameters that produce pERK rebound in CRC but not melanoma cell lines. We simulated 3-day cell cultures, setting the maximal EGFR signaling as tenfold higher in CRC vs. melanoma cells (*EGFR*_*T*_ = 1 vs. 0.1), based on *EGFR* mRNA expression from TCGA RNASeq data. For the simulations, a prototypic BRAFi was implemented, which maintains 95% target suppression. Given the stochastic nature of PSO and large number of free parameters, we ran the algorithm multiple times and selected the 10 best solutions (lowest Mean Square Error) for further analysis. The model quantitatively reproduced the pERK rebound observed in response to BRAFi treatment in CRC but not in melanoma cells, as dependent upon EGFR/RAS/CRAF signaling^18^ (Fig. 2a, b). To explore which of the three feedback circuits underlie this phenomenon, we simulated the model with each circuit turned on individually, or together (Fig. 2c). All three mechanisms were capable of producing some degree of signal rebound, but the effect was more pronounced when all three were active.

The second set of results we wished to reproduce concern the effect of mutations in core components of the MAPK cascade on the sensitivity to EGFR/MAPK inhibitors. As noted above, heightened EGFR activation mediates resistance to BRAFi treatment, as do *BRAF* amplifications.^32^ *KRAS* amplifications, and single-nucleotide substitutions, which constitutively activate KRAS (such as G12V) or MEK1 (such as F53L) also mediate resistance to combinations of BRAF, MEK, and EGFR inhibitors, though sensitivity to ERK inhibition is reportedly not affected by such mutations.^23^

Based on these findings, we ran the PSO algorithm 20 times to further calibrate the model to reproduce published mutation-treatment response profiles^23^ and predict untested mutation-treatment response pairings in *BRAF*-mutant cells. Cell growth in 3-day cultures was simulated, again assuming 95% target suppression by prototypic inhibitors (EGFRi, BRAFi, MEKi, ERKi. The results are represented as a hierarchically clustered heatmap with drug treatments on the *x*-axis, and mutational status (in addition to the *BRAF* ^V600^ mutations) on the *y*-axis (Fig. 2d). Drug treatments are ordered by relative activity, and similarly, mutations ordered by relative potency in mediating drug resistance. BRAFi treatment was effective only in cells with low expression of EGFR (and thus minimal RAS/CRAF signaling). MEKi treatment was effective in EGFR-high cells, but abrogated by *BRAF*-amplification. The BRAFi + MEKi combination was potent in all but the *MEK*-mutant cells, while ERKi treatment was effective at suppressing cell growth regardless of genetic background, consistent with published data.^21–23^

### ERK is highly sensitive to pMEK-mediated catalytic activation

We next explored why the inhibition of ERK was less sensitive to MAPK-reactivation than inhibition of MEK, by analyzing the parameter values that successfully recapitulate this difference. Focusing on the pMEK:pERK activation curve, the parameter combinations that were sampled cover a wide range of shapes (based on EC_50_ and Hill coefficients). However, the 20 parameter solutions that best reproduce the experimental data all yield an extremely sensitive pMEK-pERK relationship at low levels of pMEK, with median EC_50_ (parameter*τ*_*4*_) of 0.11 (Fig. 2e). That is, at 90% pMEK inhibition, pERK remains at 50% of maximal activity. Achieving>90% pERK reduction predicted as necessary to achieve tumor regression (Fig. S1a) would require 98% inhibition of pMEK. This provides a mechanism by which targeting ERK may be advantageous in cases of super-physiologic MAPK (MEK) signaling. Moreover, this highlights the difficulty in achieving clinically robust suppression of the pathway via RAF/MEK inhibitors, due to combined MEK-ERK signal amplification and multiple ERK-mediated negative feedback circuits.^33^

To confirm this model-predicted relationship experimentally, we quantitated published western blot measurements of pERK and pMEK in four *BRAF* ^V600E^-CRC cell lines dose-titrated with the pan-RAFi AZ628 (Colo-201 and Colo-206F, both parental and derivatives with in vitro-acquired resistance to BRAFi^32^; Table S3). The data were remarkably consistent with model predictions, supporting a high-sensitivity pMEK:pERK relationship in *BRAF* ^V600E^-CRC cells (Fig. 2e).

Our model employs an empirical (logic-based) description of the signal transduction cascade, and thus does not enable deeper interrogation of the mechanisms underlying this pMEK:pERK relationship, or an exploration of how generalizable it is to other cancer types. To do so, we extracted a sub-model of the two-step phosphorylation of ERK by pMEK from a detailed mass action kinetics-based model of the pathway (see Methods).^34^ Parameters for the kinetic rate constants and initial conditions were taken directly from the publication and based on data from HeLa cells, a *BRAF*-wild-type cervical carcinoma line. The sub-model was simulated at steady state, and the resultant pMEK vs. pERK relationship overlaid on our results (Fig. 2e). While the curve could in theory have taken on a variety of shapes depending on the values of kinetic parameters and enzyme concentrations (Fig. S1b, c), the results were again consistent with both the simulations from our model, and the experimental data. Combined, this indicates that the high-sensitivity relationship arises from the enzymatic nature of pMEK-mediated pERK activation (a biochemical amplifier),^33^ a conserved element of the pathway rather than an idiosyncratic feature of *BRAF* ^V600E^-CRC cells. This also provides a biochemical underpinning to the steep exposure–response relationship observed between the MEKi cobimetinib and pERK in vivo.^35^

### Signaling through multiple cell surface receptors abolishes the activity of BRAF inhibition in vitro

It is well established pre-clinically that signaling through EGFR can mediate resistance to BRAFi treatment, and the model successfully captures this phenomenon (Fig. 2a–d). However, clinical trials testing combinations of EGFR and BRAF inhibitors in *BRAF*-CRC have since proven disappointing.^14, 36^ One potential explanation is signaling redundancy between EGFR and alternate receptors. Many receptors are capable of activating RAS/CRAF signaling, and thus could theoretically bypass combination EGFR/BRAF blockade to activate MEK/ERK (see Fig. 1a). We tested this hypothesis by dose titrating a panel of four *BRAF* ^V600E^-CRC cell lines (HT-29, LS411N, MDST8, and SW1417) with vemurafenib (BRAFi), in the presence or absence of TGFα, HGF, and bFGF, ligands for the receptors EGFR, c-MET, and FGFR, respectively, (Fig. 2f). As expected, TGFα stimulation nullified the growth inhibitory effect of vemurafenib in all four cell lines. The effects were completely recapitulated with HGF or bFGF stimulation, implying functional redundancy between EGFR, MET, and FGFR signaling. Gene expression profiling^37^ of 11 *BRAF* ^V600E^-CRC cell lines (including 3/4 above) revealed that transcripts encoding MET and FGFR4 are expressed at levels equivalent to EGFR in these cells, as are IGFR2 and ERBB3, regulators of PI3K/Akt signaling (Fig. S2). MET and FGFR are thus capable of fulfilling the role of *RTK2*, and IGFR and ERBB3 that of *RTK3*, all representing potential mediators of resistance to EGFRi and BRAFi treatments.

### In vitro cell growth experiments allow estimation of drug–target inhibition IC_50_ values

**Fig. 3:**
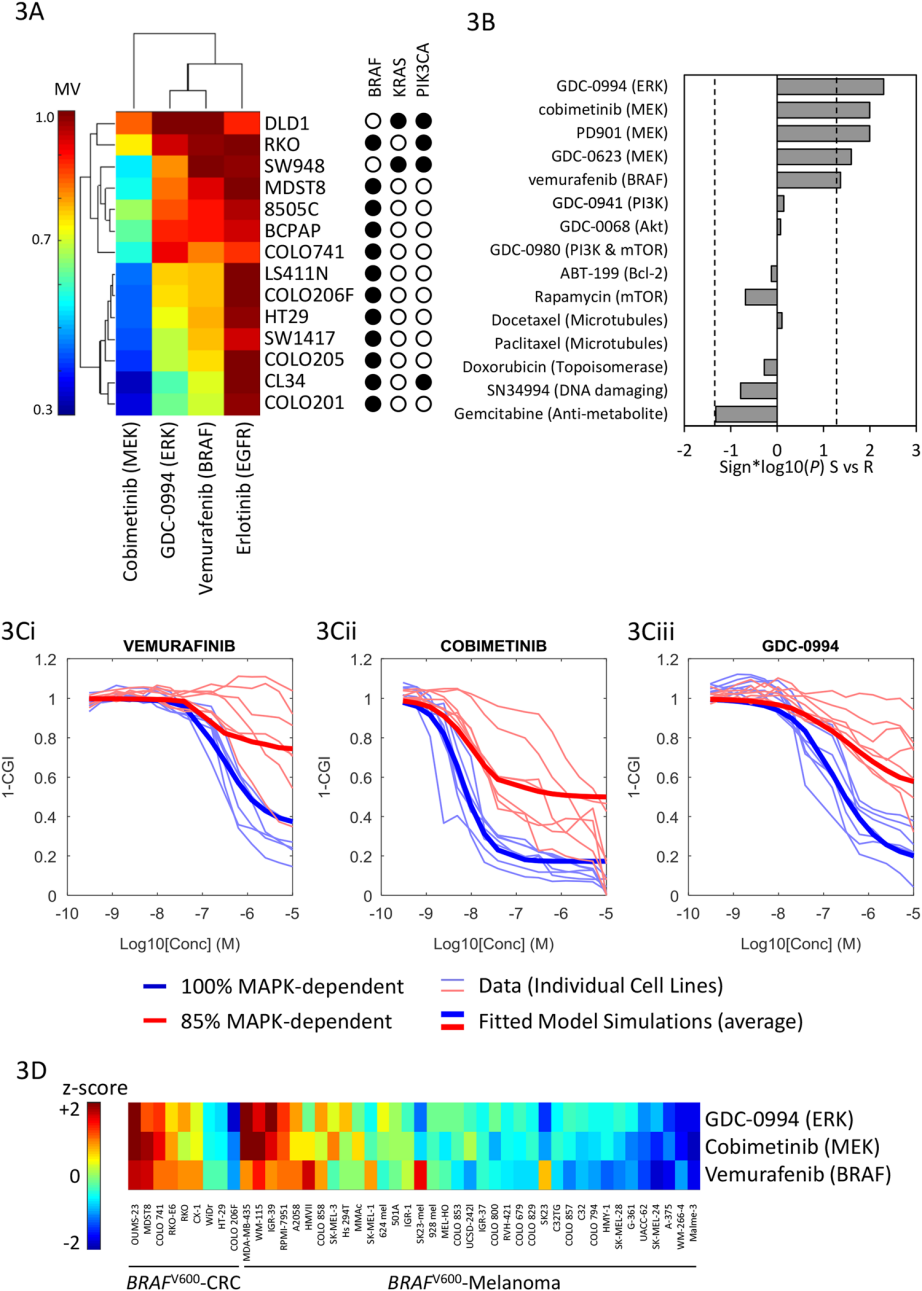
MAPKi sensitivity profiles of CRC cell lines. **a** Sensitivity (mean viability) of 14 CRC cell lines to EGFR, BRAF, MEK, and ERK inhibition. **b** Median differences in sensitivity (mean viability) between the seven sensitive (S) vs. seven resistant (R) cell lines to 15 drugs, including five MAPK pathway inhibitors, five inhibitors of other signaling pathways, and five cytotoxic chemotherapies. **c** Cell viability dose–responses of 14 cell lines to vemurafenib, cobimetinib, and GDC-0994. Cell lines are annotated as relatively MAPKi sensitive (*blue*) vs. resistant (*red*), overlying individual cell line data (*light lines*) and model simulations (*thick lines*). **c** Normalized sensitivity (*z*-scored mean viability) of nine CRC and 37 melanoma cell lines to GDC-0994 cobimetinib and vemurafenib

The analysis presented thus far was based on simulating the effect of generic inhibitors, assuming 95% target suppression in vitro. In order to simulate the effects of specific drugs, we generated cell viability dose–response curves to vemurafenib (BRAFi), cobimetinib (MEKi), and GDC-0994 (ERKi) in a panel of 14 CRC cell lines, 12 harboring *BRAF* ^V600E^ mutations and 2 with *KRAS* ^G12V^ mutations. Representing the mean viability (essentially the area under the curve) of each cell line × drug treatment as a hierarchically clustered heatmap, the cells separate into two groups corresponding to relatively MAPKi sensitive vs. resistant subsets (Fig. 3a). Notably, the two *KRAS*-mutant cells were in the resistant cluster, consistent with established knowledge about the relative sensitivity of *BRAF* vs. *KRAS*-mutants to MAPK inhibition.^38^

To assess whether the pattern was specific to MAPK pathway inhibitors, rather than generic differences in sensitivity to anti-cancer drugs, we used a cell line screening database^37^ to examine sensitivity (mean viability) of the same cell lines to 15 anti-cancer drugs, including 5 MAPK inhibitors, 5 inhibitors of non-MAPK targets (PI3K, Akt, mTOR, and Bcl-2), and 5 cytotoxic drugs. Differential sensitivities between the MAPKi-sensitive vs. resistant subsets defined by clustering (Fig. 3a) are represented in Fig 3b as directed *P*-values (rank-sum test). There were no significant differences in sensitivity to the 10 non-MAPK directed drugs, while the sensitive cluster was responsive to all RAF/MEK/ERK inhibitors tested. Differences between the cell lines are thus specific to MAPK pathway targets.

Drug-target IC_50_values and Hill coefficients for target inhibition (rather than growth inhibition) for each of the inhibitors tested were then estimated using non-linear least squares regression, treating the seven sensitive cell lines as biological replicates (i.e., drug-target IC_50_s were assumed constant across cell lines). As shown in Fig. 3c, the resulting model fits capture the data well. Median estimates for drug-target IC_50_s are represented in Table 1 (individual estimates in Table S4). The affinity of cobimetinib for MEK is estimated at roughly an order of magnitude greater than vemurafenib for BRAF^V600E^ and GDC-0994 for ERK, relatively consistent with biochemical *Ki* measurements.^28, 39, 40^ As none of the cell lines responded to erlotinib, the IC_50_ for EGFR inhibitors could not be estimated, and were thus taken from drug labels.

**Table 1:** Pharmacological properties of drugs included in the model

Parameters	Cetuximab AVG (%CV) [m/h]	Vemurafenib AVG (%CV) [m/h]	Cobimetinib AVG (%CV) [m/h]	GDC-0994 AVG (%CV) [m/h]
Ka (1/d)	–	4.5 (101) [5.3]	33 (167) [0.7]	35 (173) [0.19]
Vc/F (L)	4.5	114 (68) [0.61]	487 (51) [5.8]	171 (39) [0.07]
CL/F (L/d)	0.67	32 (33) [48]	327 (59) [15.9]	161 (43) [13.1]
V2/F (L)		–	335 (75) [1]	–
Q/F (L/d)		–	252 (72) [1]	–
IC_50_ (mg/L)	0.03	0.027 (170)	0.0032 (150)	0.13 (39)
MM (g/mol)	152 000	489	531	441
MTD (mg)	450 Q1W	960 BID	60 Q1D	400 Q1D
*mu*MTD (mg/kg)	12.5 Q1W	50 Q1D	5 Q1D	50 Q1D

*[m/h] indicates murine/human scaling factor for PK parameters, based on a 70 kg patient

### Modest differences in MAPK pathway-dependence can explain the differential sensitivity of cell lines to RAF/MEK/ERK inhibitors

To explore what biological mechanisms could underlie the differences in RAF/MEK/ERK sensitivity among cell lines, we evaluated whether any individual model parameter was capable of converting dose–response curves from the sensitive to resistant profiles. A modest decrease in cellular dependence on MAPK signaling (from 100 to 85%via reduction of the parameter *w*_OR_) recapitulated the resistant cluster dose-viability profiles to BRAFi, MEKi, and ERKi (Fig. 3c). No other single model parameter tested was capable of doing so. This suggests that even a modest activation of an alternate oncogenic pathway is sufficient to explain the significantly reduced sensitivity to MAPK inhibition in these cells.

To further explore this phenomenon, we examined sensitivity to the same BRAF/MEK/ERK inhibitors in a panel of *BRAF* ^V600^-mutant cell lines, 9 CRC and 37 melanoma.^37^ Consistent with clinical experience, the majority of melanoma cell lines were sensitive to BRAF and MEK (as well as ERK) inhibition and a fraction highly resistant, while the pattern is reversed in CRC cells (Fig. 3d). For both tissue types, responsiveness to the three drugs are highly correlated, consistent with model predictions that cellular dependence on the MAPK pathway is a critical determinant of response to BRAF/MEK/ERK inhibition. MAPK pathway dependence thus appears to be heightened and more frequent in melanoma compared to CRC. To identify potential pathways mediating MAPK inhibitor resistance, we compared differences in transcriptional profiles between the melanoma vs. CRC cell lines using pathway enrichment analysis (see Methods). CRC cells displayed heightened expression of genes related to extra-cellular matrix (ECM) organization, and pathways related to cytokine, interleukin, GPCR, Ephrin, FGF, and PI3K/Akt signaling, among others. Comparing the MAPK-sensitive vs. resistant CRC subsets from Fig. 3a, the resistant set was again enriched in transcripts related to ECM organization and collagen synthesis, as well as Rho-GTPase, Notch, Wnt, and IL-6 signaling (Table S5). While there are many caveats in inferring signaling activity from mRNA expression profiles, this suggests a multitude of pathways (aside from PI3K/Akt) may play a role in reducing cellular dependence on MAPK signaling in *BRAF* ^V600E^-CRC tumors.

### The MAPK signaling model captures in vivo xenograft growth kinetics

We next assessed the ability of the model, developed using in vitro data, to describe and predict drug activity in vivo. We established subcutaneous xenografts of HT29 cells (a *BRAF* ^V600E^-CRC model), allowed time for palpable tumors to form, and then began treatment with clinically relevant regimens of cetuximab (12.5 mg/kg Q1W), vemurafenib (50 mg/kg QD), cobimetinib (5 mg/kg QD), and GDC-0994 (50 mg/kg QD), as well as seven pairwise combinations and two triple combinations (eight animals per treatment arm, ten animals control). Tumor size was then measured every 3 days over 21 days of treatment.

**Fig. 4:**
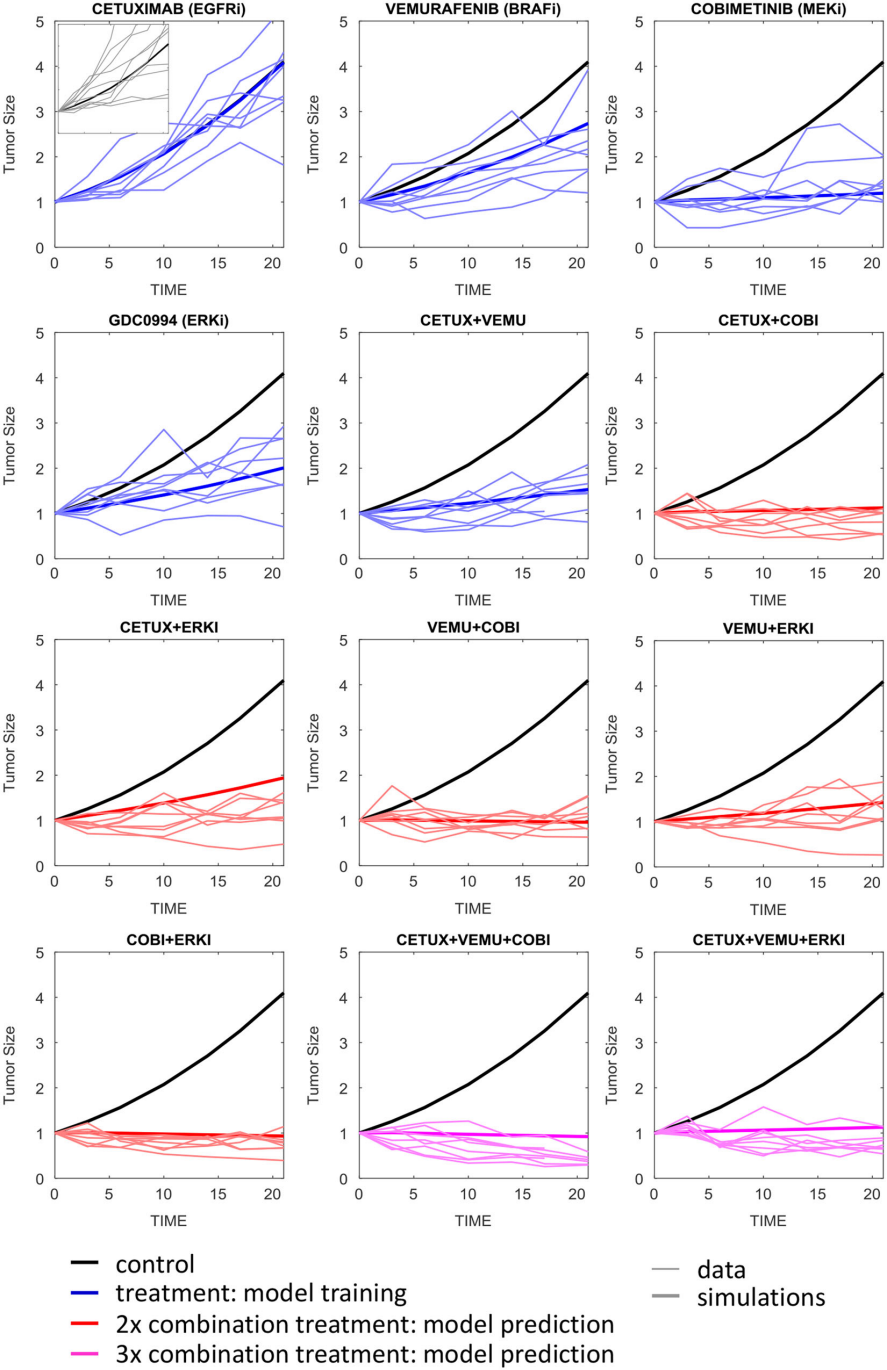
HT29 xenograft tumor growth kinetics over 21 days. Individual tumors (*thin lines*) and model simulations (*thick lines*) overlaid, with plots color-coded as: untreated control (*black*), treatment data used for model training (*blue*), and model predictions, i.e., data not used for training (*red* and *pink*, corresponding to double and triple drug combinations)

To adapt the model from an in vitro cell culture to the in vivo xenograft context, we assumed that all cellular signal transduction and drug IC_50_ parameters were conserved between the cell culture and in vivo contexts, but rates of maximal proliferation and cell death (*µ*_MAX_, *δ*_MAX_) would vary. To account for EGFR redundancy we also estimated the activity of alternate receptor signaling (*RTK2*), as ligands for alternate receptors may be expressed in vivo. Murine pharmacokinetic (PK) parameters for each drug (Table S6) were used to simulate drug exposure in the tumors, assuming a serum/tumor partition coefficient of 100%. The three in vivo parameters (*µ*_MAX_, *δ*_MAX_, *RTK2*) were then estimated by least-squares fitting to the mean tumor growth kinetics in the control and monotherapy treatment groups, as well as the cetuximab + vemurafenib combination arm (Fig. 4, parameter estimates in Table S7). The model fits the data well, consistent with our assumption that the primary difference between in vitro and in vivo experimental models are the rates of cell proliferation and death, plus alternate RTK signaling to account for the reduced activity of cetuximab. We assessed model predictivity by prospectively simulating tumor kinetics on the combination treatment arms, and overlaid the results of actual tumor data for eight select combinations (five doublets and two triplets, depicted as red and pink panels in Fig. 4). Predicted growth curves match the experimental data well, as simulations lay within the distribution of the eight replicate tumors.

### Proliferation rate, MAPK-dependence, and alternate receptor (RTK2) signaling explain the variation between in vivo tumor models

**Fig. 5:**
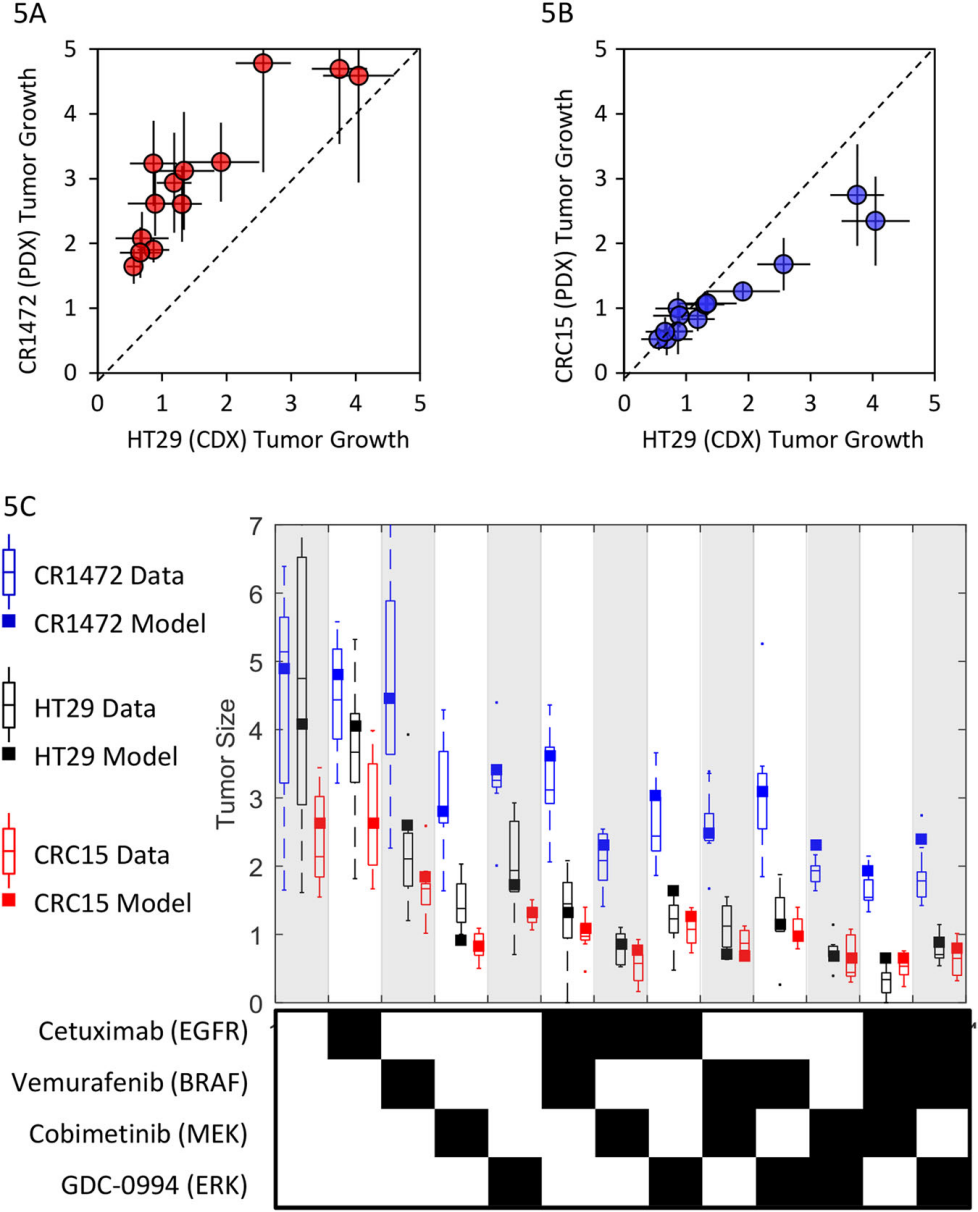
Tumor growth and treatment responses in HT29 cell-line derived xenografts vs. two PDX models, CR1472 and CRC15. **a** HT29 vs. CR1472 and **b** HT29 vs. CRC15 tumor growth over 21-days on 13 alternate treatments. **c** Model simulations and experimental observations of changes in tumor size over 21-days for the 13 treatments

All of the combinations assessed are reasonably active in the HT29 xenografts, resulting in either tumor stasis or regression. The HT29 model is thus very sensitive to MAPK-inhibition, which does not accurately reflect clinical experience. To explore more clinically relevant models, we performed the same in vivo experiment using two patient-derived xenografts (PDX models CR14172 and CRC15), which are expected to more closely mirror clinical responses than do cell line-derived xenografts (CDX).^25^ Focusing on the 21-day tumor size changes (Fig. 5a), the untreated CR1472 tumors grow slightly faster than the HT29 xenografts, with approximately 4.5 vs. 4-fold increases in volume over the three-week period. The CR1472 tumors were also considerably less responsive to all therapies (all 12 treatments lay above the CR1472 vs. HT29 diagonal), and even the triple combinations failed to achieve tumor stasis. In contrast, the CRC15 PDX tumors grew more slowly than the HT29 (2.7-fold), but both models responded similarly to drug treatments (Fig. 5b). In fact, while the magnitude of treatment responses varied significantly between tumor models, the rank order of treatment effects were very consistent, with Spearman’s correlation coefficients of 0.86 between the HT29 vs. CR1472 and 0.94 between the HT29 vs. CRC15 (*P* *<* 10^−4^ for both comparisons).

We used the computational model to explore which biological parameters were necessary and sufficient to convert an HT29-like tumor to the two alternate PDX tumors. After performing a Local Parameter Sensitivity Analysis (Table S8) and assessing the effect of multiple mutations, protein expression changes, and cellular and pharmacological properties on simulated HT29 tumor growth (Fig. S3), we found the refractory CR1472 response profile could be matched by adjusting just three model parameters. First, by increasing the *RTK2* activity (i.e., non-EGFR receptor signaling) from a median value of 3.9% to 39% that of EGFR, thereby reducing the sensitivity to cetuximab combinations. Second, by modestly increasing the proliferation rate (*µ*_MAX_) from 0.23 to 0.28 per day, thus increasing tumor growth. Third, by either decreasing the dependence of growth on MAPK(*w*_OR_) from 100% to 78%, or by decreasing the drug tumor partition coefficient from 100% to 6%. That is, the response profile of a tumor with modestly reduced dependence on the MAPK pathway is very similar to that with a drastically reduced tumor drug concentration. Because the model fits were better with the reduced MAPK hypothesis (*r* ^2^ = 0.95 vs. 0.85; Fig. S3c **)**, and it seems physiologically unlikely that drug penetration into the CR1472 tumors would be systematically reduced to this extent compared to the HT29 xenografts (20-fold), dependence on alternate (non-MAPK) pathways seems a more plausible explanation for the reduced responsiveness.

The CRC15 tumor growth kinetics and drug response profile could be matched precisely by tuning just a single parameter from the HT29 model, reducing the proliferation rate (*µ*_MAX_) from 0.23 to 0.17 per day. When we attempt to estimate the MAPK-dependence parameter (*w*_OR_) for this model, an optimal value of 96% is found, close enough to 100% to conclude that these tumors are solely dependent on MAPK signaling, as are the HT29 cell line-based xenografts.

We thus have two PDX tumor models with very different drug response patterns, one of which is completely refractory to all combination treatments (CR1472), and one which achieves either tumor stasis or regression on the various combination regimens (CRC15) (Fig. 5c). However, it is unclear to what extent either of these PDX models reflects human CRC tumors. The experiments could be repeated in a panel of PDX models representing the breadth of genetic diversity of *BRAF* ^V600E^-CRC tumors to estimate population-level response rates to the combinations. While such experiments have been conducted^26, 41^ the approach seems impractical to implement as a platform tool in drug development. As an alternative approach, we used available clinical data to translate the computational model from xenografts to patients, and represent the diversity of *BRAF* ^V600^-CRC tumors in silico.

### Clinical translation and prediction of novel drug combination efficacy

**Fig. 6:**
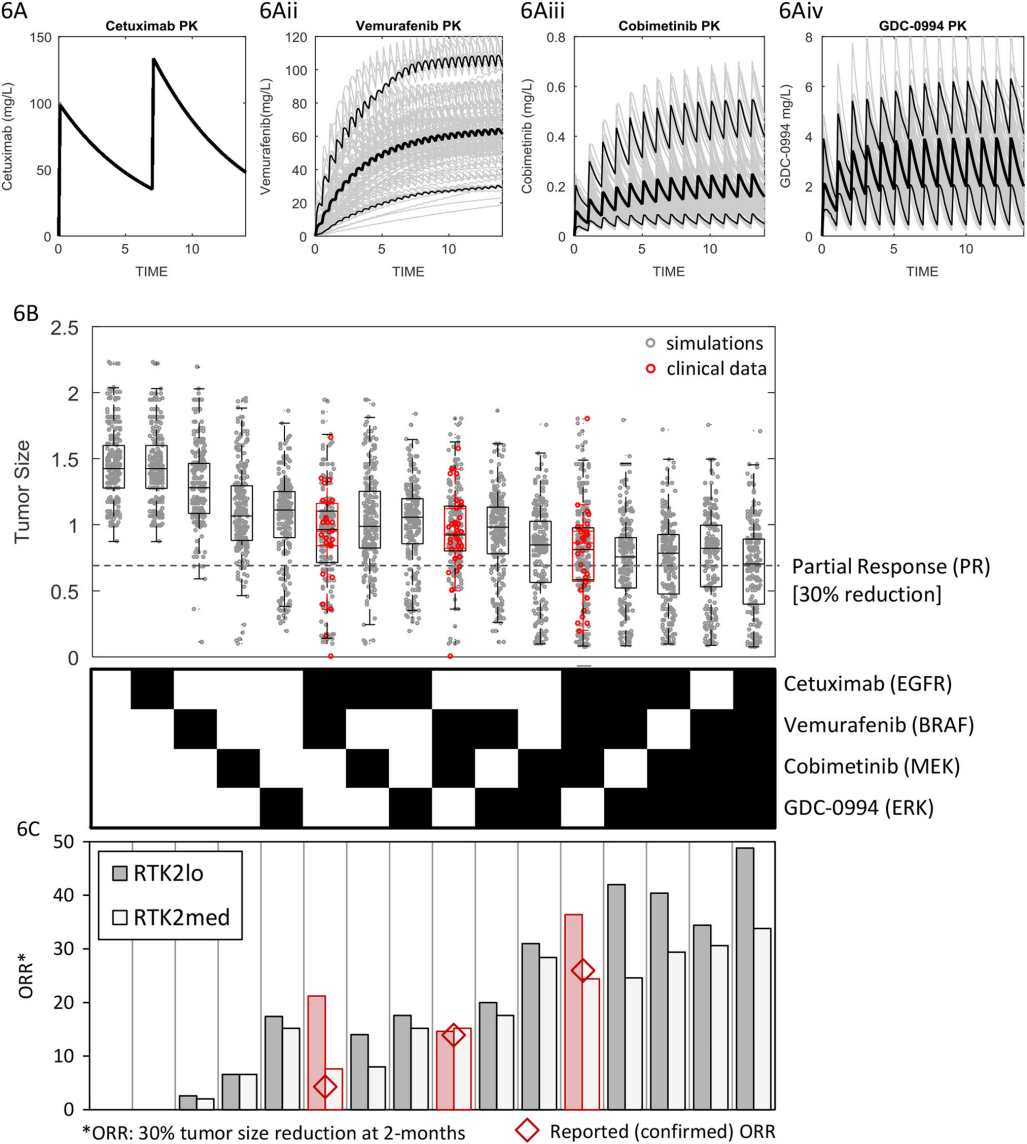
Simulated pharmacokinetics and tumor responses on 16 drug treatment regimens. **a** Simulated serum drug concentrations for cetuximab, vemurafenib, cobimetinib, and GDC-0994 dosed at clinical regimens. Median, 5 and 95-percentiles are shown in *black lines*. **b** Relative changes in tumor size following two cycles of treatment (8 weeks) on all 16 possible combinations of the four drugs, for prevalence weighted model simulations (*gray*) and clinical data (*red*). **c** Simulated ORRs for baseline tumors (*RTK2*lo), and those with *RTK2* set at 15% that of EGFR (*RTK2*med)

Two changes enabled the translation of the model from murine xenografts to the clinical context. First, mouse PK parameters were replaced with human counterparts, including population-level variability. Population PK models for cetuximab and vemurafenib were taken from Pharmacology Review sections of BLA/NDA fillings (www.accessdata.fda.gov), cobimetinib from published literature,^42^ and GDC-0994 developed from data collected as part of a Phase 1clinical trial^43^ (summary PK parameters in Table 1, and covariance matrices in Table S9). These models were used to simulate concentration time-courses of each drug at clinical dosing regimens across a population (Fig. 6a).

The second, and more challenging step was to alter the model to reflect the cell biology of a clinically relevant patient population rather than a xenograft. Quantitative information on the molecular and cellular differences between these settings is lacking. However, published patient-level tumor responses in *BRAF* ^V600E^-mutant CRC are available from Phase 1b clinical trials testing combinations of vemurafenib + cetuximab,^14^ the alternate BRAF and MEK inhibitors dabrafenib + trametinib,^16^ and dabrafenib + trametinib + panitumumab (an EGFRi).^19^ Under the assumption that the alternate BRAF, MEK, and EGFR inhibitors are clinically equivalent,^44, 45^ we used the data from these three studies to constrain our simualtions, and predict clinical responses to GDC-0994-containing regimens.

To do so, we first selected 16 model parameters representing variable or uncertain quantitative biology for randomization. These correspond to expression levels and basal enzymatic activity of key protein species, feedback circuits, and degree of MAPK-pathway dependency. A virtual cohort of 1000 tumors was created by Monte Carlo sampling across log-normal distributed parameter values (Table 2), and tumor kinetics simulated in response to treatment with all 16 possible combinations of the four drugs (cetuximab, vemurafenib, cobimetinib, and GDC-0994) at single-agent maximum tolerated dose (MTD) regimens (Table 1). For cobimetinib and GDC-0994 this consisted of daily dosing with 21/7-day on/off cycles, while cetuximab and vemurafenib were dosed continuously (weekly and twice daily). Change in tumor size at 8 weeks of therapy, as per RECIST criteria^46^ was then used to classify simulated treatment responses.

**Table 2:** Model parameters selected for variation across the virtual population

Parameter	Description	Mean	Log_10_(variance)
BRAFt	maximal BRAF activity (i.e., gene amplification)	1	1
CRAFt	maximal CRAF activity (i.e., gene amplification)	1	1
dmax	Cellular death rate	0.04 per day	0.1
G13	Feedback Gain: ERK-EGFR feedback	0.5	1
Gdusp	Feedback Gain: ERK-DUSP feedback	0.5	1
Gspry	Feedback Gain: ERK-SPRY feedback	0.5	1
MEKb	Minimal MEK activity (i.e., activating mutation)	0.01	2
MEKt	Maximal MEK activity (i.e., gene amplification)	1	1
PI3Kb	Minimal PI3K activity (i.e., activating mutation)	0.05	2
PI3Kt	Maximal PI3K activity (i.e., gene amplification)	1	1
RASb	Minimal RAS activity (i.e., activating mutation)	0.05	1
RASt	Maximal RAS activity (i.e. gene amplification)	1.5	1
RTK1t	Maximal EGFR activity	0.25	2
RTK2t	Maximal RTK2 activity	0.1	2
wOR	MAPK pathway dependence (quantitative OR gate)	1	0.1
wRAS	RAS-PI3K activation strength (quantitative OR gate)	0.9	0.1

Patient-level response data (waterfall plots) from the three clinical trials were digitized and binned into RECIST categories (Table 3), and quadratic optimization used to match the response distributions between the simulations and clinical data by assigning a prevalence weight (PW), or statistical probability, to each virtual tumor in the population.^47^ Monte Carlo resampling from the prevalence weighted virtual cohort (Table S10) was then performed to generate a virtual population, and simulate clinical responses to the 16 treatments arms. Simulated tumor size changes closely matched the clinical data (Fig. 6b), and provided predictions for the remaining twelve treatment arms which have not been tested clinically.

**Table 3:** Distribution of patient-level tumor response data from published clinical studies in *BRAF* ^V600E^-CRC

% Change from baseline	CETUX + VEMU% (*N*)	BRAFi + MEKi% (*N*)	EGFRi + BRAFi + MEKi% (*N*)
100+	0	0	0
50:100	3.8 (1)	2.6 (1)	2.9 (1)
20:50	11.5 (3)	10.5 (4)	0
0:20	30.8 (8)	26.3 (10)	11.4 (4)
−30:0	30.8 (8)	47.4 (18)	48.6 (16)
−50:−30	7.7 (2)	10.5 (4)	20.0 (8)
−100:−30	15.4 (4)	2.6 (1)	17.1 (6)
Calculated ORR	23.1 (6/26)	13.1 (5/38)	37.1 (13/35)
Reported ORR	4 (1/27)	12 (5/43)	26 (9/35)

*EGFRi, BRAFi, and MEKi were panitumumab, dabrafenib, and trametinib. Data from refs 14, 16, 19

From the proportion of virtual tumors which regressed by 30% or more, we calculated the ORR for each treatment arm. Simulations predict more-than additive activity for many of the combinations (Table S11), in particular the vemurafenib + cobimetinib-containing regimens due to co-targeting of signaling redundancies (BRAF plus EGFR/CRAF). There is, however, a discrepancy between the results of ORRs calculated from the maximal change in tumor size at 8 weeks, and the “confirmed” ORRs, shown in Fig. 6b and noted in Table 3. This discrepancy presumably represents tumors that initially shrank by greater than 30% over the first 8-week cycle, but then subsequently began to regrow over the second cycle of therapy such that they would be classified as “unconfirmed” (i.e., transient) responses. Notably, this discrepancy appears for both EGFRi treatment arms, but not BRAFi + MEKi, suggesting that activation of alternate RTK signaling is mediating resistance to EGFR inhibition, and consistent with our cell line data demonstrating functional redundancy between EGFR, MET, and FGFR signaling (Fig. 2e). We, therefore, examined how increasing *RTK2* (non-EGFR) signaling affects simulated response rates. With *RTK2* activity increased to 15% that of pretreatment EGFR activity, the simulated ORRs for all four treatment arms closely matched the confirmed ORRs reported in the publications (Fig. 6c).

### GDC-0994+/- cobimetinib are predicted to be the most efficacious single and double agent treatments

**Fig. 7:**
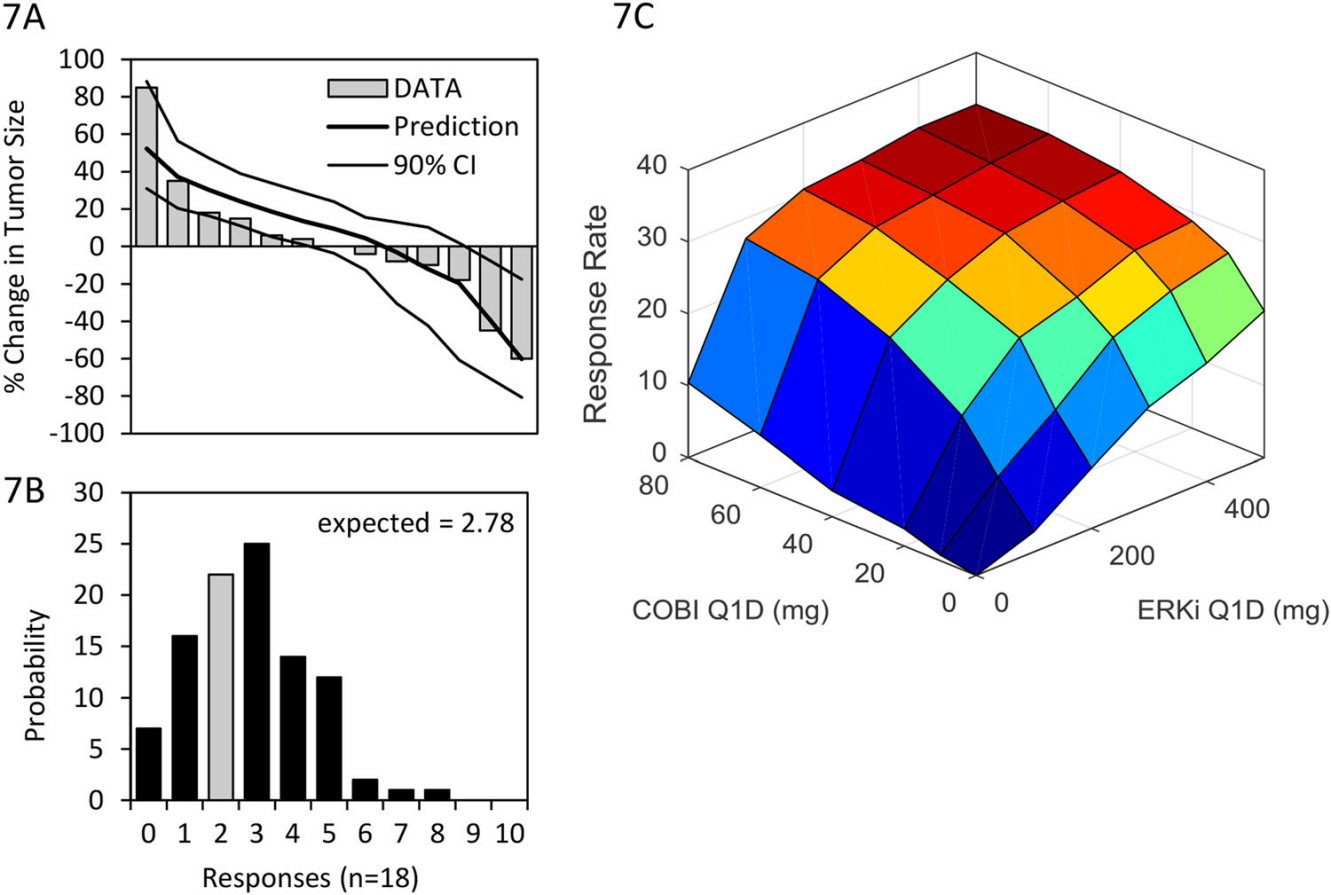
Clinical trial simulations and data for GDC-0994 monotherapy, and combination with cobimetinib. **a** Simulated tumor size changes (waterfall plots), and data from 13 evaluable patients treated with GDC-0994. **b** Simulated distribution of expected responses for an 18 patient clinical trial, *gray bar* indicating clinical results (2/18). **c** Simulated ORR to combinations of cobimtenib and GDC-0994

GDC-0994 treatment was predicted to yield the highest monotherapy response, at approximately 17% ORR (independent of the degree of*RTK2* signaling), compared to 8% for cobimteinib, 3% for vemurafenib, and 0% for cetuximab. We simulated 8-week tumor size changes (waterfall plots) on GDC-0994 monotherapy for 100 replicate trials, estimating 90% confidence intervals based on the underlying biological and pharmacological variability. Subsequently, as part of a Phase I study of GDC-0994 monotherapy (NCT01875705), 18 *BRAF* ^V600E^-mutant CRC patients were treated, of which 13 had evaluable tumors.^43^ The measured changes in tumor size match model predictions remarkably well for the prevalence-weighted virtual tumors (Fig. 7a), with statistically indistinguishable predicted vs. measured distributions (*P* = 0.71; rank-sum test). In contrast, the simulated distribution of the virtual cohort from which the virtual population was sampled (i.e., without clinical data-based prevalence weighting), was significantly different than the clinical result (*P* = 0.039; Fig. S4a). Incorporation of clinical data via prevalence weighting was thus necessary to generate accurate predictions.

Given a predicted ORR of 17% across the virtual population, we would expect 2.8 responses of the 18 patients entering treatment. Two confirmed partial responses were observed, consistent with model predictions (*P* = 0.25; binomial test comparing 2/18% vs. 17%, Fig. 7b). The simulated response rate in the 1000 tumor virtual cohort without prevalence weighting (51% ORR) was again significantly different than the clinical result (*P* = 2.5 × 10^−5^), further confirming that inclusion of clinical data into the model via the prevalence weighting approach was necessary in making accurate predictions. Predictive accuracy of the model was found to increase progressively with each clinical data set included in the weighting, the greatest value emanating from the triple combination study (Fig. S4c, d). While the three clinical data sets were found to add differing degrees of predictive power, no pattern was discernable from the small number available. In summary, inclusion of the clinical data from EGFRi, BRAFi, and MEKi treatments into a pre-clinically constructed model was necessary and sufficient to accurately predict clinical tumor responses to GDC-0994 monotherapy.

The combination of cobimetinib + GDC-0994 is predicted to be the most active doublet combination, with a predicted ORR of 32% at the single-agent MTDs of both. As this regimen may not be tolerated, we simulated responses to the combination of cobimetinib + GDC-0994 over a range of doses (10–80 mg and 100–500 mg Q1D, respectively,; Fig. 7c). Predictions indicate synergistic (more than additive) activity of the drugs, particularly at lower doses. However, even at the highest combination dose schedule, predicted response rates of 33% are far lower than the 68% ORR observed with the vemurafeninb + cobimetinib combination in *BRAF* ^V600E^-melanoma.^48^ We thus used the model to explore the molecular or cellular features limiting clinical tumor responses, and prospectively assess how these could be overcome.

### Clinical responses to MEK/ERK inhibitors could be increased via predictive biomarker-based patient stratification or alternate combination therapies

**Fig. 8:**
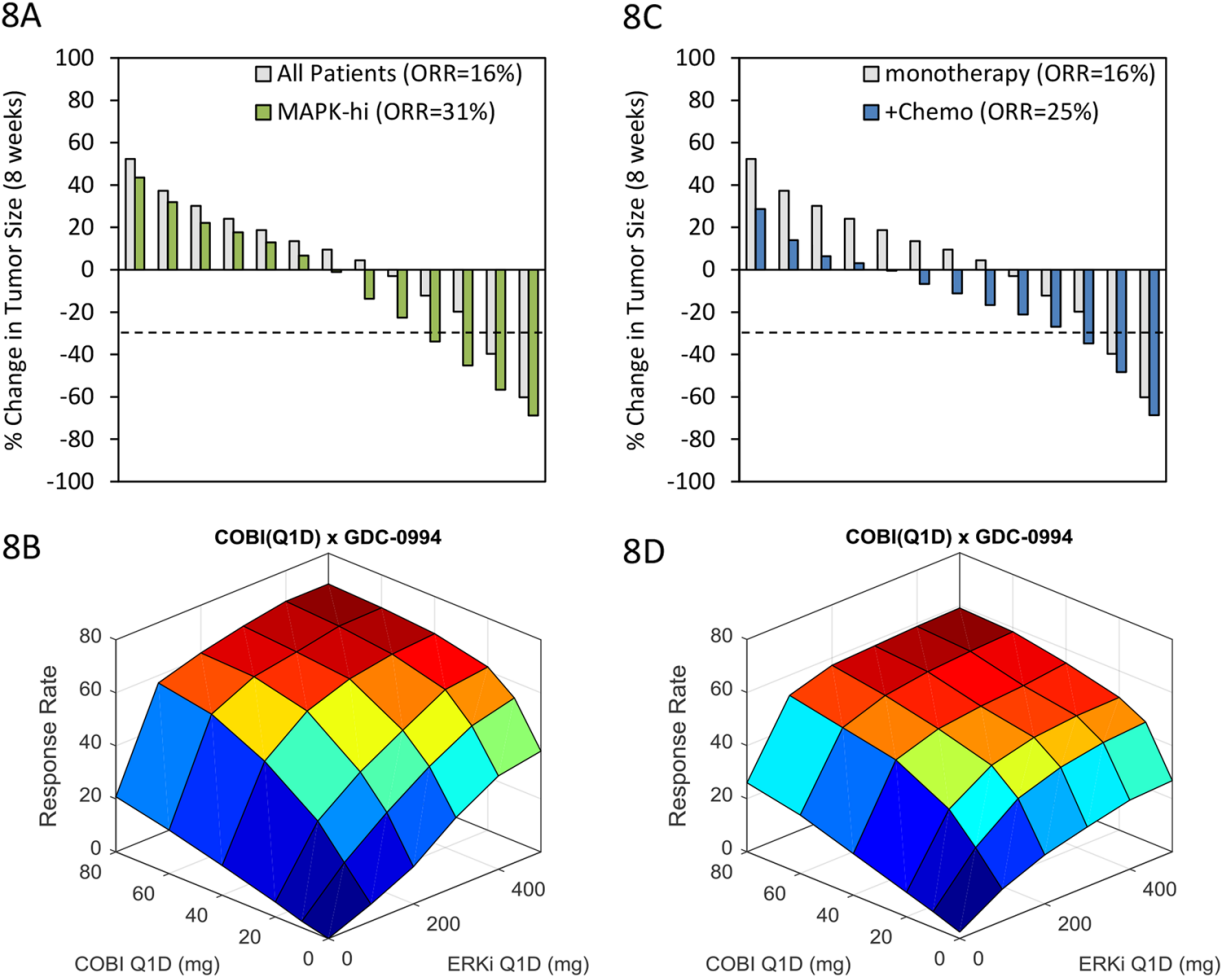
Enhancement of GDC-0994/cobimetinib responses via biomarker stratification or combination with additional anti-cancer agents. Simulated tumor responses (waterfall plots), and cobimetinb x GDC-0994 combination surface responses for **a**, **b** patients selected with greater than the 50-percentile MAPK-dependence, and **c**, **d** combination with another anti-cancer agent that increases the rate of cell death by 10% independent of MAPK signaling

To examine the molecular and cellular features (biomarkers) associated with drug response/resistance, we built a multivariate linear regression model linking 27 model variables (16 specified in Table 2 plus 11 PK parameters specified in Table S9) to simulated tumor growth responses to the 16 treatments, as described.^49^ Examination of the normalized regression coefficients revealed the most dominant predictors of response across treatments are MAPK pathway dependence (*w*_OR_) and the maximal rate of cell death (*δ*_MAX_) (Fig. S5). That is, virtual tumors that respond well to treatment are highly dependent on MAPK signaling for growth rather than other pathways (such as PI3K/Akt), and have increased apoptotic sensitivity. This suggests that response rates could be improved using a predictive biomarker to prospectively select patients with heightened MAPK signaling dependence for treatment, or by combining MEK/ERK inhibitors with anti-cancer agents targeting alternate mechanisms of cell survival (i.e., alternate oncogenic pathway inhibitors, cytotoxic chemotherapy, anti-apoptotic inhibitors, or immuno-therapies). We used the model to change these two clinical scenarios. First, we simulated the effect of selecting virtual patients with MAPK-dependence (*w*_OR_) in the top 50-percentile of the population, and predicted results of the GDC-0994 monotherapy clinical trial (Fig. 8a) and GDC-0994 + cobimetinib combination dose response surface (Fig. 8b) in this sub-population. Second, we simulated the same clinical scenarios, but combined treatment with an unspecified drug that modestly increases the rate of cell death by 10%, independently of MAPK signaling (Fig. 8c, d). Under both scenarios, the ORR approximately doubled for both GDC-0994 monotherapy (31% and 25%) and the GDC-0994 + cobimetinib combination (68% and 59%). Thus, with appropriate clinical strategies, response rates in at least sub-populations of *BRAF* ^V600E^-CRC patients could possibly approach that achievable in *BRAF* ^V600E^-melanoma.

## Discussion

We have described the step-wise development of a mechanism-based model of the MAPK signaling network in *BRAF* ^V600^-mutant CRC. The model links cellular biochemistry and genetics to in vitro cell growth, in vivo tumor kinetics in CDX and PDX models, and ultimately clinical tumor responses. In contrast to empirical PK/PD models, which are largely descriptive in nature,^50^ incorporating mechanistic details of the molecular and cell biology enabled accurate translational predictions. Specifically, the model predicts and emphasizes the importance of a hypersensitive relationship between pMEK and ERK activity. This prediction was subsequently validated using quantitative western blot data from *BRAF*-CRC cell lines^32^ and through simulations of a published mass action kinetics-based model of the MAPK pathway.^34^ Furthermore, this finding is consistent with theoretical and experimental work demonstrating that the MAPK pathway demonstrates “ultrasensitivity”^51^ and functions as a “negative feedback amplifier”.^33^ This hypersensitive relationship underlies the steep exposure-response relationship between cobimetinib and pERK suppression,^35^ the ability of MAPK hyper-activating mutations to cause MEKi resistance, and the increased in vitro responsiveness to ERKi vs. MEKi in this context.^20–23^ In vivo, the model described tumor kinetic responses to single agent treatments and predicted combination effects. We identified minimal cellular differences between the three in vivo xenograft models (proliferation rate, alternate (non-EGFR) RTK expression, and MAPK-dependence) that suffice to capture the differential tumor growth response patterns between them. Finally, we generated a virtual patient population using published clinical data on BRAF, MEK, and EGFR antagonists, which accurately predicted population level-tumor responses to single agent treatment with the ERKi GDC-0994, and projected strategies to increase the single agent responses.

MEK inhibitors have been tested extensively in many solid tumors, both as monotherapies and in combination with cytotoxic drug regimens.^52^ Yet despite the wealth of pre-clinical data posing MAPK as a critical oncogenic pathway, clinical activity has been minimal outside of melanoma.^53^ In addition to the well-established robustness of the MAPK network due to feedback circuits and pathway cross-talk,^54^ our results provide two critical explanations for this. First, the hypersensitive pMEK:pERK relationship necessitates near-complete target suppression for antitumor activity, and this may be difficult to achieve with tolerable doses of MEK inhibitor monotherapies. That is, MEK lies upstream of a signal amplification step and is embedded within multilayered feedback control circuits, making it a particularly challenging target. ERK inhibition, and particularly the ERK/MEK inhibitor combination is less susceptible to pathway reactivation, and thus easier to sustain thorough target suppression.

**Fig. 9:**
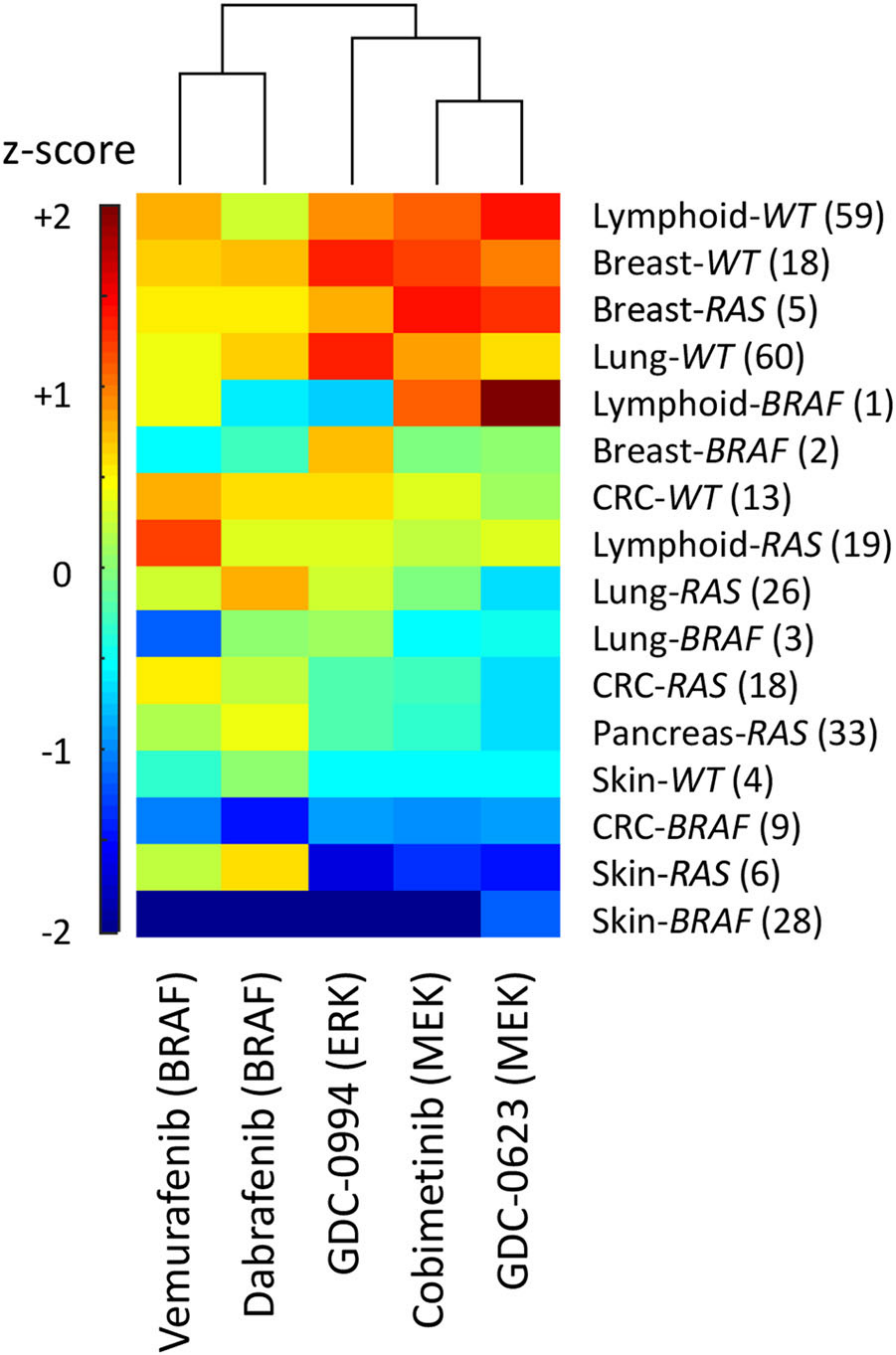
Relative sensitivity (*z*-scored mean viability) to BRAF, MEK, and ERK inhibition by indication. Cancer cell lines were separated by tissue type and mutational status (RAS = KRAS, HRAS or NRAS mutant, RAF = BRAF-mutant, WT = RAS/RAF wild type), and median values computed for each

Secondly, cellular dependence on the MAPK cascade appears to be highly variable across tumors. Our results suggest that the majority of *BRAF* ^V600^-mutant CRC tumors, despite constitutive signal flux through the MAPK pathway, contain at least some clones that are intrinsically or adaptively reliant on other oncogenic signaling modules, and, therefore, capable of expansion under the therapeutic pressure of MEK/ERK inhibition. To expand on this concept more systematically, we analyzed the relative sensitivity of 329 cell lines to multiple BRAF/MEK/ERK inhibitors, classified by tissue source and RAF/RAS mutational status (Fig. 9). Melanomas (skin) are indeed an outlier, as both *BRAF* and *NRAS*-mutant cells are particularly sensitive to all forms of MAPK inhibition. Increasing the activity of BRAF/MEK/ERK inhibitors in CRC and other indications will thus necessitate the use of predictive biomarkers for patient selection, and/or combinations with drugs targeting orthogonal pathways.

In line with this, recent Phase 1 studies in *BRAF* ^V600E^-CRC have reported that addition of the topoisomerase inhibitor irinotecan to BRAFi + MEKi combination increased activity^55^ (ORR of 35% compared to 12%) (ref. 16), as did the PI3K inhibitor alpelisib to BRAFi + EGFRi treatment^56^ (ORR of 18% compared to 4% (ref. 14) or 17% (ref. 36) for the doublet). The main challenge in pursuing such multi-drug combination strategies is managing toxicity. While predictive biomarkers of MAPK inhibitor sensitivity could be used to increase clinical activity, the development of such diagnostics remains elusive, as intuitive measurements such as pERK often have little predictive value.^57, 58^ More complex, multivariate assays will likely be necessary to achieve clinically meaningful patient selection.

Within *BRAF* ^V600E^-CRC tumors, a few therapeutic principles can be drawn. If tumors dependent upon MAPK signaling could be pre-identified, ERKi (GDC-0994)-containing regimens, particularly in combination with cobimetinib (MEKi) are predicted to be particularly active. EGFR inhibition provides additional activity by suppressing the activation of MAPK and other effector pathways, both those engaged directly by EGFR and downstream of Ras, such as PI3K/Akt.^59^ However, as revealed by both our analyses and clinical experience in CRC,^60^ multiple cell surface receptors are capable of filling the role of *RTK2* and functionally substituting for EGFR, notably MET and the FGFR-family. If signaling through a panel of these receptors could be effectively quantified and monitored, receptor antagonists could be rationally employed. Alternatively, Ras or C-Raf inhibitors (many in early development) could potentially substitute for the multitude of receptor antagonists.

The nature and significance of alternate, non-MAPK pathways remains both a black box in our model, and a hindrance to treatment of this disease. Gene expression analyses suggest a number of pathways could be involved, notably signaling through ECM components such as Integrins, as well as Rho-GTPases, Wnt/β-catenin, Notch, and PI3K/Akt originally represented in the model structure. Signaling through the PI3K/Akt pathway has been shown to mediate adaptive resistance to MAPK pathway inhibition in *BRAF*-CRC xenograft models.^61, 62^ While the limited clinical data reveal no relationship between PI3K pathway mutations and MAPKi resistance,^16^ gene expression analyses reveal that approximately 1/3 of *BRAF* ^V600^-CRC tumors show heightened PI3K/Akt/mTOR signaling uncorrelated with *PIK3CA* mutations.^63^ Alternatively, phenotype switching could account for intrinsic or adaptive loss of MAPK-signaling dependence, as has been observed in *BRAF* ^V600E^- melanomas.^64, 65^ This is associated epithelial-mesenchymal transition (EMT) in carcinomas, and notably, “ECM organization”, one of the top pathways associated with MAPKi resistance form our transcriptome analyses, includes TGF-β pathway ligands (TGFB1, BMP1/4, and LTBP1), drivers of EMT.^66^ Ultimately, this black box represents fundamental gaps in our understanding of cell signaling networks and their role in cancer progression.^67^

Computational models can help bridge the divide between pre-clinical data and clinical strategy. In this case, clinical data sets were available for inhibitors of closely connected targets (EGFR, BRAF, and MEK) in the same indication (*BRAF* ^V600E^-CRC), and this data was necessary to statistically constrain the clinical simulations and make accurate predictions. Predicting responses to drugs in new therapeutic indications, or for targets lacking related clinical data would be much more challenging. However, many drug programs have some clinical precedents for related targets or pathways. Our results reveal that with fairly minimal and biologically plausible parameter tuning, a single model structure, based on state-of the-art literature review, is capable of translating results from in vitro cell culture, to in vivo tumor xenografts, to clinical predictions.

## Methods

### Model structure

Cellular signal transduction from cell surface receptors, via the MAPK and PI3K pathways, to the surrogate signaling output (S6), as well as feedback circuits regulating pathway output were encoded using quantitative logic-based algebraic equations as described.^68, 69^ While detailed mass action kinetics-based models of these signaling cascades exist in the literature,^33, 34, 70, 71^ these have been developed to capture short term (minutes to hours) signaling dynamics, rather clinically relevant time scales (days to weeks). At longer time scales, signal transduction events will be at quasi-steady state and can thus be represented algebraically. We thus developed a system of differential-algebraic equations wherein signal transduction events are described by algebraic Hill functions, while drug PKs, signaling feedback, and tumor growth are described using ordinary differential equations (ODEs), with a goal of capturing the essential biology while limiting mathematical complexity and simulation time. Algebraic equations are executed in tandem at each time step of the ODE solver (SUNDIALS, IDA), as per the order listed below. In the following equations, EGFR serves as *RTK1*, DUSP as *FB1*, SPRY as *FB2*, MYCas *FB3*, and FOXO as *FB4*.

$$\begin{array}{ccccc}\\ RTK1 =\kern-8pt & RTK{1_b}{\rm{ + }}\left( {RTK{1_t} - RTK{1_b}} \right) \cdot \left( {1 - {G_{13}} \cdot \displaystyle \frac{{F{B_3}^{kFB3}}}{{{\tau _{FB3}}^{kFB3} + F{B_3}^{kFB3}}}} \right) \cdot \hfill \\ & \left( {1 - \displaystyle \frac{{RTK1{i^{ki1}}}}{{{\tau _{i1}}^{ki1} + RTK1{i^{ki1}}}}} \right)\hfill \\ \end{array}$$

$$\begin{array}{ccccc}\\ RTK2\,{\rm{ = }}\,RTK{2_b}{\rm{ + }}\left( {RTK{2_t} - RTK{2_b}} \right) \cdot \left( {1 - {G_{23}} \cdot \displaystyle \frac{{F{B_3}^{kFB3}}}{{{\tau _{FB3}}^{kFB3}{\rm{ + }}F{B_3}^{kFB3}}}} \right) \\ \end{array}$$

$$\begin{array}{ccccc}\\ RTK3\,{\rm{ = }}\,& \kern-8pt RTK{3_b}{\rm{ + }}\left( {RTK{3_t} - RTK{3_b}} \right) \cdot \left( {1 - {G_{33}} \cdot \displaystyle \frac{{F{B_3}^{kFB3}}}{{{\tau _{FB3}}^{kFB3}{\rm{ + }}F{B_3}^{kFB3}}}} \right) \cdot \hfill \\ & \left( {1 - {G_{34}} \cdot \displaystyle \frac{{F{B_4}^{kFB4}}}{{{\tau _{FB4}}^{kFB4}{\rm{ + }}F{B_4}^{kFB4}}}} \right)\hfill \\ \end{array}$$

$$\begin{array}{ccccc}\\ RAS\,{\rm{ = }}\,& \kern-10pt RA{S_b}{\rm{ + }}\left( {RA{S_t} - RA{S_b}} \right) \cdot \left( {\displaystyle \frac{{{{\left( {RTK1 + RTK2} \right)}^{k1}}}}{{{\tau _1}^{k1} + {{\left( {RTK1 + RTK2} \right)}^{k1}}}}} \right) \cdot \hfill \\ & \left( {1 - \displaystyle \frac{{F{B_2}^{kFB2}}}{{{\tau _{FB2}}^{kFB2} + F{B_2}^{kFB2}}}} \right)\hfill \\ \end{array}$$

$$\begin{array}{ccccc}\\ BRAF\,{\rm{ = }}\,& \kern-10pt BRA{F_b}{\rm{ + }}\left( {BRA{F_t} - BRA{F_b}} \right) \cdot \left( {\displaystyle \frac{{RA{S^{k2}}}}{{{\tau _2}^{k2} + RA{S^{k2}}}}} \right) \cdot \hfill \\ & \left( {1 - \displaystyle \frac{{BRAF{i^{ki2}}}}{{{\tau _{i2}}^{ki2} + BRAF{i^{ki2}}}}} \right)\hfill \\ \end{array}$$

$$\begin{array}{ccccc}\\ MEK\,{\rm{ = }}\,& \kern-10pt ME{K_b}{\rm{ + }}\left( {ME{K_t} - ME{K_b}} \right) \cdot \left( {\displaystyle \frac{{{{\left( {BRAF{\rm{ + }}CRAF} \right)}^{k3}}}}{{{\tau _3}^{k3}{\rm{ + }}{{\left( {BRAF{\rm{ + }}CRAF} \right)}^{k3}}}}} \right) \cdot \hfill \\ & \left( {1 - \displaystyle \frac{{MEK{i^{ki3}}}}{{{\tau _{i3}}^{ki3} + MEK{i^{ki3}}}}} \right)\hfill \\ \end{array}$$

$$\begin{array}{ccccc}\\ ERK\,{\rm{ = }}\,& \kern-10pt ER{K_b}{\rm{ + }}\left( {ER{K_t} - ER{K_b}} \right) \cdot \left( {\displaystyle \frac{{ME{K^{k4}}}}{{{\tau _4}^{k4}{\rm{ + }}ME{K^{k4}}}}} \right) \cdot \left( {1 - \displaystyle \frac{{F{B_1}^{kFB1}}}{{{\tau _{FB1}}^{kFB1}{\rm{ + }}F{B_1}^{kFB1}}}} \right) \cdot \hfill \\ & \left( {1 - \frac{{ERK{i^{ki4}}}}{{{\tau _{i4}}^{ki4}{\rm{ + }}ERK{i^{ki4}}}}} \right)\hfill \\ \end{array}$$

$$CRAF\,{\rm{ = }}\,CRA{F_b}{\rm{ + }}\left( {CRA{F_t} - CRA{F_b}} \right) \cdot \left( {\frac{{RA{S^{k5}}}}{{{\tau _5}^{k5} + RA{S^{k5}}}}} \right)$$

$$PI3K = PI3{K_b} + \left( {PI3{K_t} - PI3{K_b}} \right) \cdot \left( {\frac{{{{\left( {{w_{RAS}} \cdot RAS{\rm{ + }}RTK3} \right)}^{k7}}}}{{{\tau _7}^{k7}{\rm{ + }}{{\left( {{w_{RAS}} \cdot RAS{\rm{ + }}RTK3} \right)}^{k7}}}}} \right)$$

$$AKT\,{\rm{ = }}\,AK{T_b}{\rm{ + }}\left( {AK{T_t} - AK{T_b}} \right) \cdot \left( {\frac{{PI3{K^{k8}}}}{{{\tau _8}^{k8} + PI3{K^{k8}}}}} \right)$$

$$S6\,{\rm{ = }}\,S{6_b}{\rm{ + }}\left( {S{6_t} - S{6_b}} \right) \cdot \left( {\frac{{{{\left( {{w_{OR}} \cdot ERK{\rm{ + }}\left( {1 - {w_{OR}}} \right) \cdot AKT} \right)}^{k6}}}}{{{\tau _6}^{k6} + {{\left( {{w_{OR}} \cdot ERK{\rm{ + }}\left( {1 - {w_{OR}}} \right) \cdot AKT} \right)}^{k6}}}}} \right)$$

First order transit compartments were used to account for time delays in functional activity of the three ERK and AKT-mediated feedback circuits (FB_1_ through FB_4_) and between cytoplasmic signal transduction and cell growth:

$$\frac{{dF{B_1}}}{{dt}}\,{\rm{ = }}\,{r_1} \cdot \left( {ERK - F{B_1}} \right)$$

$$\frac{{dF{B_2}}}{{dt}}\,{\rm{ = }}\,{r_2} \cdot \left( {ERK - F{B_2}} \right)$$

$$\frac{{dF{B_1}}}{{dt}}\,{\rm{ = }}\,{r_3} \cdot \left( {ERK - F{B_3}} \right)$$

$$\frac{{dF{B_4}}}{{dt}}\,{\rm{ = }}\,{r_4} \cdot \left( {AKT - F{B_4}} \right)$$

$$\frac{{dT{D_1}}}{{dt}} = {r_5} \cdot \left( {S6 - T{D_1}} \right)$$

Cell number (tumor size) over time was described using quantitative logic-based differential equations, wherein cell signaling output (via the time delayed effector *TD*_*1*_) regulates cell proliferation, the rate of cell death is held constant, and tumor kinetics described using the logistic growth equation.

$$\frac{{d{\rm{CELLS}}}}{{dt}}\,{\rm{ = }}\,\left( {{\mu _{{\rm{MAX}}}} \cdot \left( {\frac{{TD_1^{{\rm{kg}}}}}{{\tau _{\rm{g}}^{{\rm{kg}}}{\rm{ + }}TD_1^{{\rm{kg}}}}}} \right) - {\delta _{{\rm{MAX}}}}} \right) \cdot \left( {1 - \frac{{{\rm{CELLS}}}}{{{V_{{\rm{MAX}}}}}}} \right) \cdot {\rm{CELLS}}$$

Drug PKs were described using standard one or two compartment ODE-based models, with absorption from the gut for the small molecules:

$$\frac{{dEGFR{i_{{\rm{blood}}}}}}{{dt}}\,{\rm{ = }}\, - k{e_1} \cdot EGFR{i_{{\rm{blood}}}}$$

$$\frac{{dBRAF{i_{{\rm{gut}}}}}}{{dt}}\,{\rm{ = }}\, - k{a_2} \cdot BRAF{i_{{\rm{gut}}}}$$

$$\frac{{dBRAF{i_{{\rm{blood}}}}}}{{dt}}\,{\rm{ = }}\,k{a_2}\frac{{{F_2}}}{{{V_2}}}BRAF{i_{{\rm{gut}}}} - k{e_2} \cdot BRAF{i_{{\rm{blood}}}}$$

$$\frac{{dMEK{i_{{\rm{blood}}}}}}{{dt}}\,{\rm{ = }}\,k{a_3}\frac{{{F_3}}}{{{V_3}}}MEK{i_{{\rm{gut}}}} - k{e_3} \cdot MEK{i_{{\rm{blood}}}} - \frac{Q}{{{V_3}}}MEK{i_{{\rm{blood}}}}{\rm{ + }}\frac{Q}{{{V_{3B}}}}MEK{i_{C3B}}$$

$$\frac{{dMEK{i_{C3B}}}}{{dt}}\,{\rm{ = }}\,\frac{Q}{{{V_3}}}MEK{i_{{\rm{blood}}}} - \frac{Q}{{{V_{3B}}}}MEK{i_{C3B}}$$

$$\frac{{dERK{i_{{\rm{gut}}}}}}{{dt}}\,{\rm{ = }}\, - k{a_4} \cdot ERK{i_{{\rm{gut}}}}$$

$$\frac{{dERK{i_{{\rm{blood}}}}}}{{dt}}\,{\rm{ = }}\,k{a_4}\frac{{{F_4}}}{{{V_4}}}ERK{i_{{\rm{gut}}}} - k{e_4} \cdot ERK{i_{{\rm{blood}}}}$$

Local (tumor) concentrations of the drugs were described using tumor partitioning coefficients, set at 1 as default:

$$RTK1i\,{\rm{ = }}\,{p_1} \cdot RTK1{i_{{\rm{blood}}}}$$

$$BRAFi\,{\rm{ = }}\,{p_2} \cdot BRAF{i_{{\rm{blood}}}}$$

$$MEKi\,{\rm{ = }}\,{p_3} \cdot MEK{i_{{\rm{blood}}}}$$

$$ERKi\,{\rm{ = }}\,{p_4} \cdot ERK{i_{{\rm{blood}}}}$$

The model equations were implemented in MATLAB SimBiology, and all simulations performed in MATLAB R2015b.

The mass action kinetics based-model of the MEK-ERK two-step enzymatic cascade was implemented as described in:^34^

$$ERK\mathop{\longleftrightarrow}\limits^{{f{\rm{1/r1}}}}pERK\mathop{\longleftrightarrow}\limits^{{f2/r2}}ppERK$$

$$\frac{{d[ERK]}}{{dt}} = {f_1} \cdot ppMEK \cdot ERK - {r_1} \cdot pAse \cdot pERK$$

$$\frac{{d[pERK]}}{{dt}}\,{\rm{ = }}\,{f_1} \cdot ppMEK \cdot ERK - {r_1} \cdot pAse \cdot pERK$$

$$\frac{\displaystyle {d[ppERK]}}{\displaystyle {dt}}\,{\rm{ = }}\,{f_2} \cdot ppMEK \cdot pERK - {r_2} \cdot pAse \cdot ppERK$$

The fractional active ERK (*ppERK*/*ERK*_*T*_) as a function of *ppMEK* was solved at steady state:

$$\frac{{ppERK}}{{ER{K_T}}}\,{\rm{ = }}\,\frac{{{f_1} \cdot {f_2} \cdot ppME{K^2}}}{{{r_1} \cdot {r_2} \cdot pAs{e^2}{\rm{ + }}{f_1} \cdot {r_2} \cdot pAse \cdot ppMEK{\rm{ + }}{f_1} \cdot {f_2} \cdot ppME{K^2}}}$$

Parameters values were taken from the paper’s supplement: *f*_*1*_ = 5.3 × 10^7^/s, *f*_*2*_ = 1.9 × 10^7^/s, *r*_*1*_ = 5.6 × 10^6^/s*, r*_*2*_ = 3.6 × 10^6^/s, *pAse* = 1 × 10^7^ mol/cell, *MEK*_*T*_ = 2.2 × 10^7^ mol/cell, *ERK*_*T*_ = 2.1 × 10^7^ mol/cell. Normalized to total MEK, *pAse* = 0.45 and *ERK*_*T*_ *= *0.95.

### Parameter estimation

Free model parameters (28; defined in Table S2) were estimated via PSO with 100 particles and iteration limit of 5000, implemented using the MATLAB Global Optimization Toolbox. The objective function was defined as mean squared error between simulations of pERK dynamic response data over 48 h (ref. 18) or in vitro cell growth over 72 h (ref. 23). As the majority of model parameters were non-identifiable, the PSO algorithm was run 20 times (Parameter estimates, distributions, and model errors are also reported in Table S2). This took approximately 30 h to complete on a desktop computer, resulting in parameter estimates that generally span the entire bounded range but with similar, and reasonably good model errors (average MSE = 4.6%, ranging between 1.3 to 9.4%). All subsequent analyses are based on average simulations across the 20 parameter sets.

*BRAF* ^V600^ mutations were simulated by fixing the minimal enzymatic activity of BRAF (*BRAFb*) = 0.9 (i.e., 90% maximal activity). Melanoma cells were defined as having a maximal EGFR expression/activity value (*RTKt*) = 0.1, and CRC as *RTKt* = 1.BRAF and KRAS-amplified cells were defined by setting maximal BRAF and KRAS activities, *BRAFt* and *RASt* = 5, respectively, (vs. 1 for wild-type), BRAF, KRAS, and MEK mutants by setting the minimal value of respective enzyme activities *BRAFb*, *KRASb*, and *MEKb* to 0.9, respectively, (vs. 0 for WT). For the initial fitting to in vitro data, generic inhibitors were assumed to achieve 95% target suppression.

Drug-target IC_50_s were estimated using cell viability (Cell Titer Glo) dose–response data. With all cellular parameters fixed, the IC_50_(*tau*_*i*_) and Hill (*k*_*i*_) coefficients for each drug were estimated by least-squares minimization between model simulations and mean response profiles of the seven sensitive *BRAF*-CRC cell lines.

In vivo HT29 xenograft cell proliferation (*µ*_MAX_) and death (*δ*_MAX_) rates, and *RTK2* activity were estimated using 21-day tumor growth kinetics of the HT29 xenografts. The parameters were estimated by least-squares minimization between model simulations and mean kinetics of eight replicate experiments (animals). Fitting the PDX models CR1472 and CRC15 was also done using least squares minimization, implemented with the MATLAB *nlinfit* function.

### Sensitivity analyses

Local parameter sensitivity coefficients were calculated based around HT29 xenograft baseline parameter values (*P*), wherein each parameter was changed by + 10% (Δ*P*; for those with baseline values of 0, these were increased to 0.1, and for those with baseline values of 1, these were decreased to 0.9 and sensitivities calculated as compared to 0.99), and relative change in tumor size (Δ*T/T*) after 21 days simulated for all 13 treatment conditions in Figs. 4 and 5.

$$LPSC = \frac{{\Delta T}}{T}/\frac{{\Delta P}}{P}$$

This was performed for all 20 parameter sets, with average values reported in Table S8. To assess the effects of focused molecular perturbations on in vivo tumor growth (compared to baseline HT29 xenografts; Fig. S3a) parameters were changed as following; Melanoma, *RTK1* = 0.1; RTK2-hi, *RTK2* = 1; PI3K/AKT-Dependence, *w*_OR_ = 0.5; BRAF-amp/KRAS-amp/MEK-amp, *BRAFt*/*KRASt*/*MEKt* = 10; MEK-mut, *MEKb* = 1; Partiton-LO, *Pi* = 0.05.

### Monte Carlo simulations

For population PK simulations, PK model variables were randomized via Monte Carlo sampling across log-normal distributions as defined by:

$${P_i}\,{\rm{ = }}\,THET{A_i} \cdot {e^{ET{A_i}}}$$

Where in *THETA* is the population mean value of PK parameter *P*_*i*_, and *ETA* is a random variable, with mean 0 and variance defined from the covariate matrices *OMEGA*, as per standard population PK modeling notation.^72^ *THETA* and *OMEGA* values for each drug were taken from FDA Clinical Pharmacology reviews and provided in Table S9.

Similarly, randomization of cellular parameters to create the virtual population was done via Monte Carlo sampling across log-normal distributions as described,^73^ with mean and variances as defined in Table 3, and 1000 randomized parameter sets given in Table S10.

### Prevalence weighting of virtual tumor data

Quadratic programming was used to assign PWs to the virtual tumors by matching simulated changes in tumor size to the clinical data in Table 3, using the approach described in:^47^

$$\mathop {{\min }}\limits_x \left[ {\frac{1}{2}x \cdot H \cdot {x^T}} \right]\,{\rm{such}}\,{\rm{that}}\,{A_{{\rm{eq}}}} \cdot x\,\,{\rm{ = }}\,\,{b_{{\rm{eq}}}}$$

Where *x* = prevalence weight (PW) vector, *H* *=* identity matrix, *A*_eq_ = simulated tumor size changes, and *b*_eq_ = actual tumor size changes reported in three clinical trials. For clinical trial simulations, virtual tumors are sampled with frequencies proportional to the PW (the final column in Table S10).

### Cell viability experiments

Cell lines were obtained from the Genentech cell line repository and maintained in RPMI 1640 medium supplemented with 10% FBS and 2 mM l-glutamine. Compounds were obtained from the Genentech compound management as 10 mM dimethyl sulfoxide stock solutions. For cell viability assays, cells were plated in normal growth medium at 1000–2000 cells per well in 384-well clear-bottom black plates. The following day, compounds were serially diluted starting at indicated concentrations, then added to cells in quadruplicates. Ninety-six hours following compound addition, CellTiter-Glo Luminescent Cell Viability reagent (Promega) was added per manufacturer’s protocol. For studies with growth factors, 20 nM TGF-alpha, 100 ng/mL HGF, or 10 ng/mL FGF was added during plating of cells and at time of drug treatment.

### Xenograft experiments

GDC-0994 was formulated in 40% PEG400 (polyethylene glycol 400)/60% [10% HP-b-CD (hydroxypropyl-beta-cyclodextrin)]. Cobimetinib was prepared as a suspension at various concentrations in methyl cellulose tween. Vemurafenib was formulated in Klucel™ hydroxypropylcellulose. Cetuximab was diluted in PBS. GDC-0994, GDC-0973, and vehicle control dosing solutions were prepared once a week, while Vemurafenib was formulated every other day, stored at 4–7 °C, and mixed well by vortexing before dosing.

All xenograft studies were performed as previously described.^74^ Briefly, human cancer cells or tumor pieces were used for implantation, to generate the HT-29 CDX or the PDX models CRC15 (Genendesign, Inc.) and CR1742 (Crown Bioscience, Inc.). HT-29 cells were grown in normal growth media (RPMI 1640 with l-glutamine and 10% fetal calf serum), harvested and implanted subcutaneously into the right flank of female NCR nude mice (6–8 weeks old) obtained from Taconic (Cambridge City, IN) weighing an average of 24–26 g. The CRC15 and CR1742 studies were run at Genendesign, Inc. and Crown Bioscience, Inc. in Balb/c nude mice. Only animals that appeared to be healthy and that were free of obvious abnormalities were used for each study. Tumor volumes were determined using digital calipers (Fred V. Fowler Company, Inc.) using the formula (*L* × *W* ^2^)/2. Mice were weighed twice a week using a standard scale.

### Gene expression and pathway enrichment analysis

Gene-level RNASeq data (RPKM) available for the MAPKi-sensitive (COLO 206 F, HT-29, SW-1417, COLO-205, CL-34, COLO 201) vs. resistant (DLD-1, RKO,SW-948, MDST8, COLO-741) CRC cell line lines, and for *BRAF* ^V600^-mutant CRC (*n* = 9) vs. melanoma (*n* = 28) cell lines were taken from (ref. 37). Differential expressed genes (rank-sum *P*-values < 0.05 or 0.01, respectively, and |fold-change| > 3) were analyzed for enrichment of Reactome pathways (www.reactome.org) via Binomial tests using PANTHER (www.pantherdb.org).^75^

## Electronic supplementary material

Figure S1

Figure S2

Figure S3

Figure S4

Figure S5

HillEQ.m

VPop_Simulator_fixed.m

Supplementary Tables S1-S11

MAPK_model.sbioproj

Supplementary Materials

Supplementary Materials
