# Erratum: Clinical responses to ERK inhibition in *BRAF*^V600E^-mutant colorectal cancer predicted using a computational model

**DOI:** 10.1038/s41540-017-0026-z

**Published:** 2017-09-04

**Authors:** Daniel C. Kirouac, Gabriele Schaefer, Jocelyn Chan, Mark Merchant, Christine Orr, Shih-Min A. Huang, John Moffat, Lichuan Liu, Kapil Gadkar, Saroja Ramanujan

**Affiliations:** Genentech Research & Early Development, 1 DNA Way, South San Francisco, CA 94080 USA

**Erratum to:** *npj Systems Biology and Applications* (2017); doi:10.1038/s41540-017-0016-1; Published 2 June 2017

After online publication of this article, the authors noticed an error in Supplemental File 7 (*VPop_Simulator.m*).

The file as has been corrected and replaced with *VPop_Simulator_fixed.m*.

The authors apologize for any inconvenience caused.

