# Supplementary figures and images for: Clinical responses to ERK inhibition in BRAFV600E-mutant colorectal cancer predicted using a computational model
Source: NPJ Syst Biol Appl. 2017 Jun 2;3:14. doi: 10.1038/s41540-017-0016-1 (PMC5460205; doi:10.1038/s41540-017-0016-1)

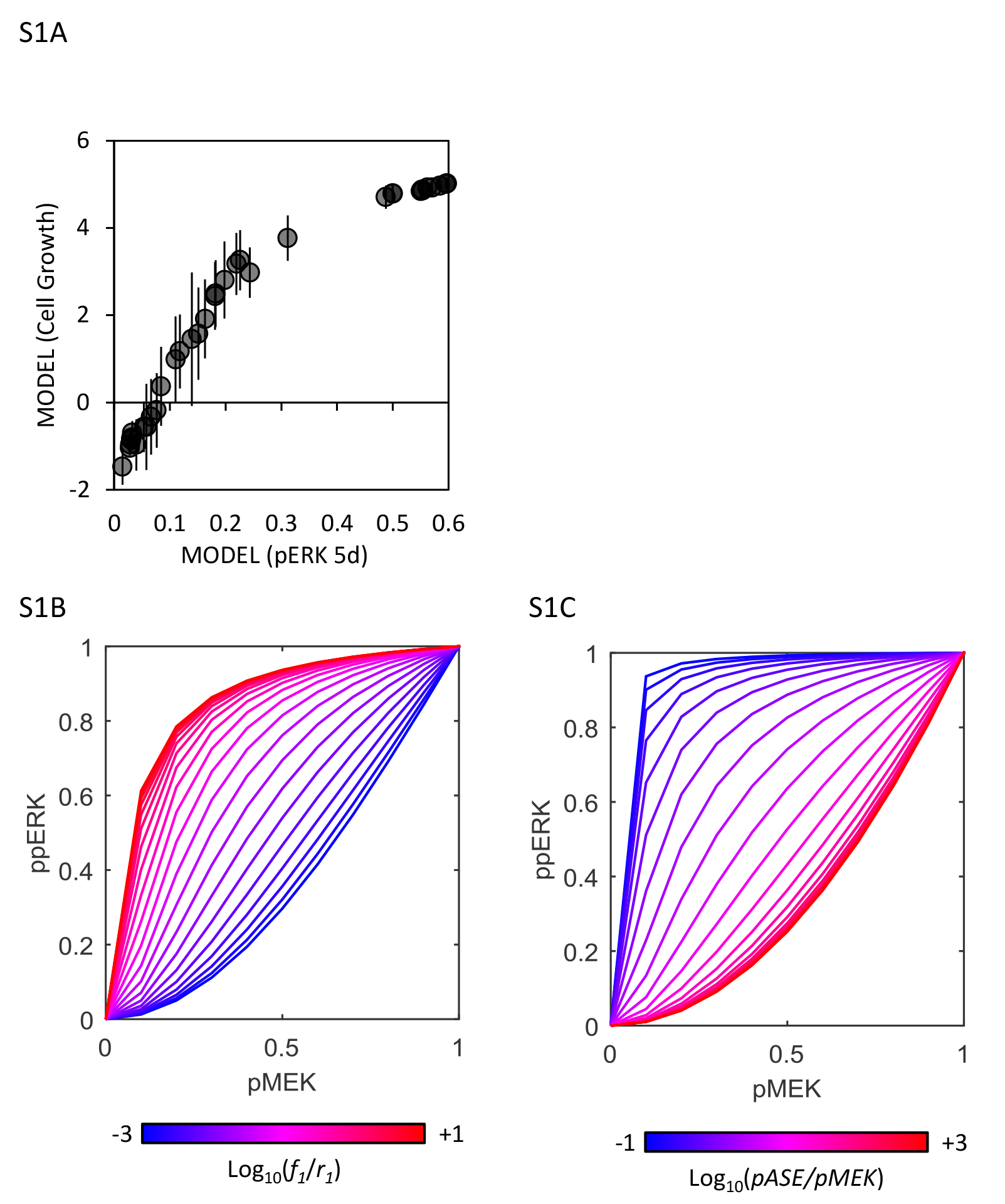

Supplement: Supplementary file 1 — Figure S1 [file 41540_2017_16_MOESM1_ESM.tif]

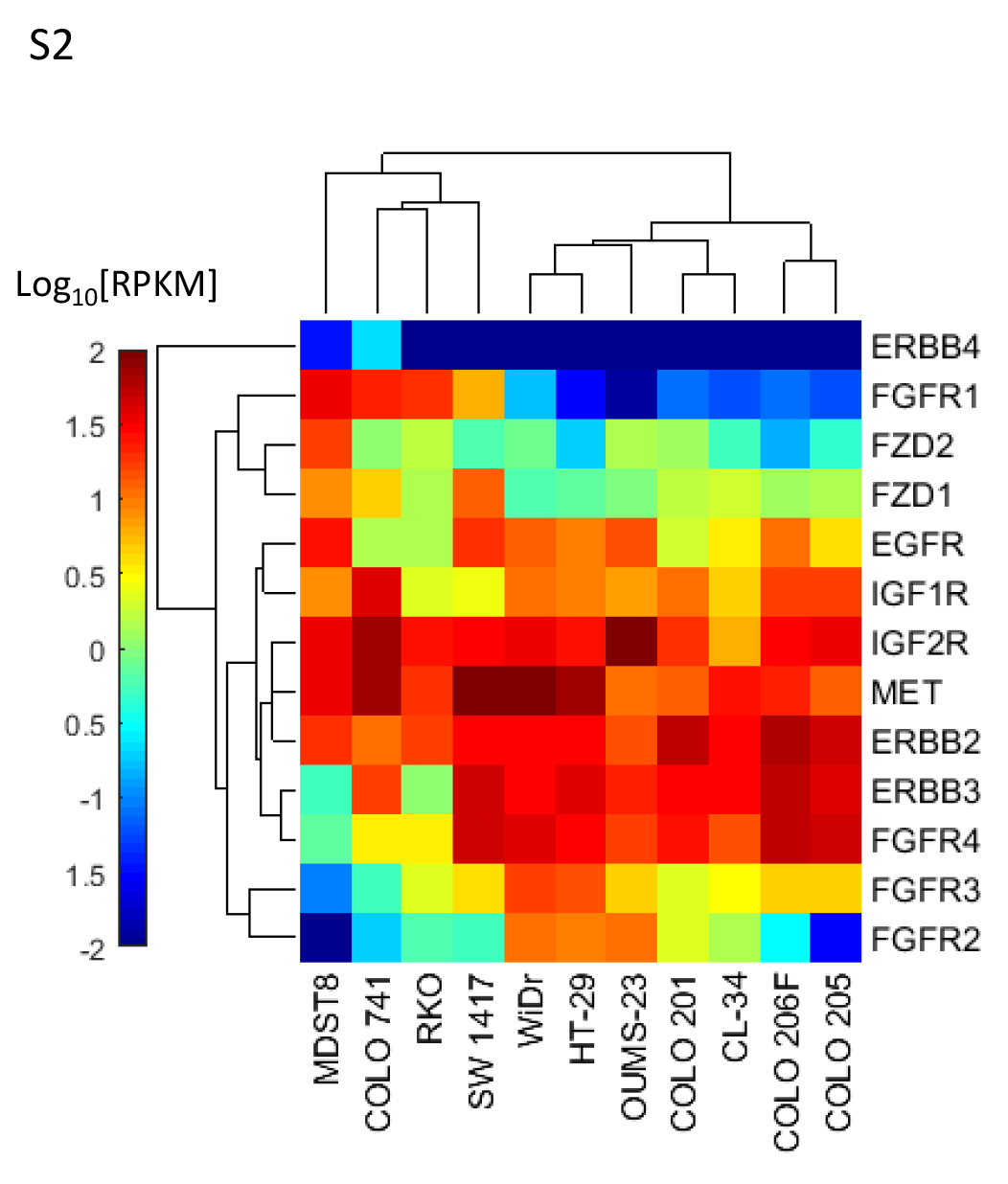

Supplement: Supplementary file 2 — Figure S2 [file 41540_2017_16_MOESM2_ESM.tif]

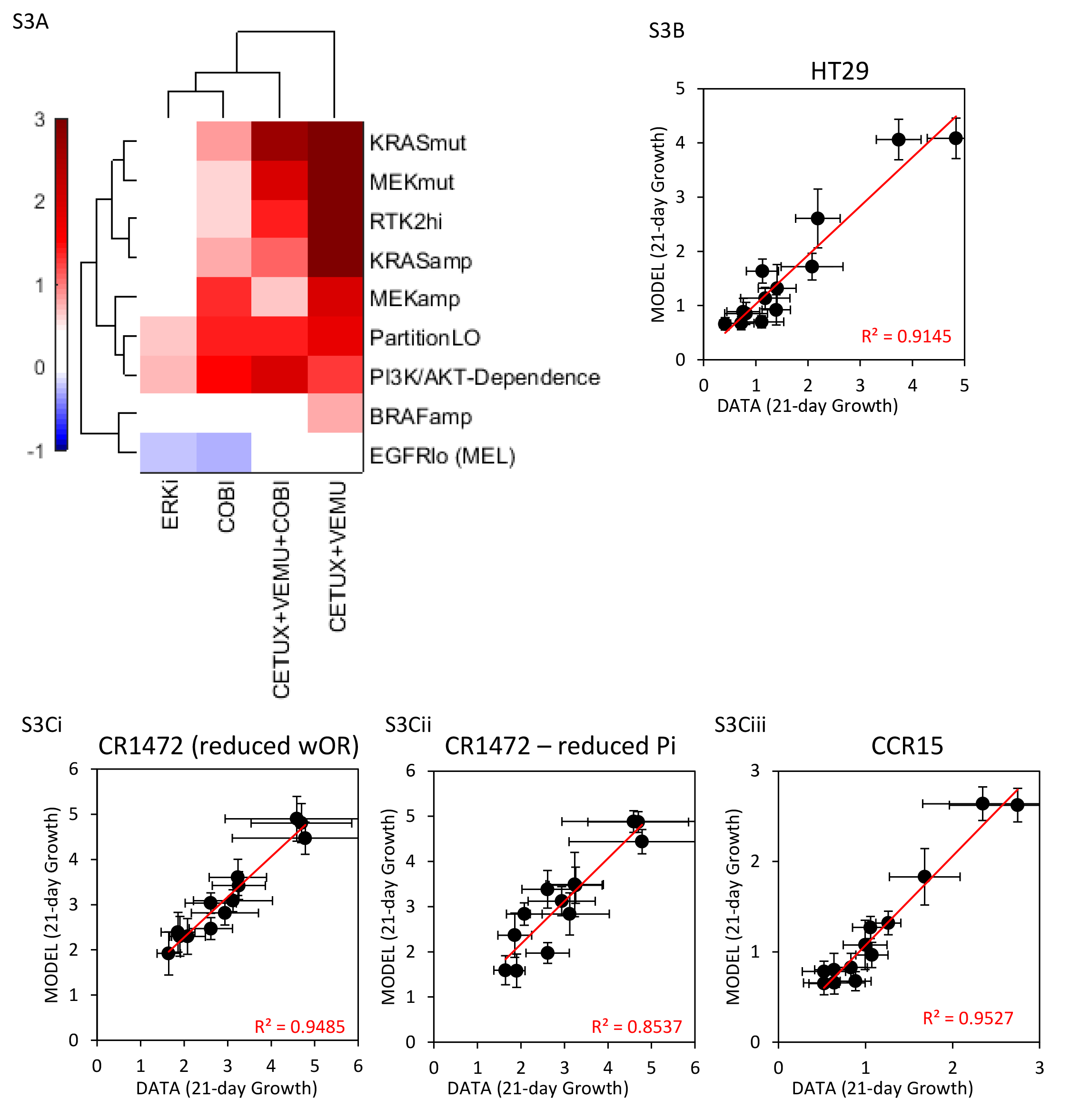

Supplement: Supplementary file 3 — Figure S3 [file 41540_2017_16_MOESM3_ESM.tif]

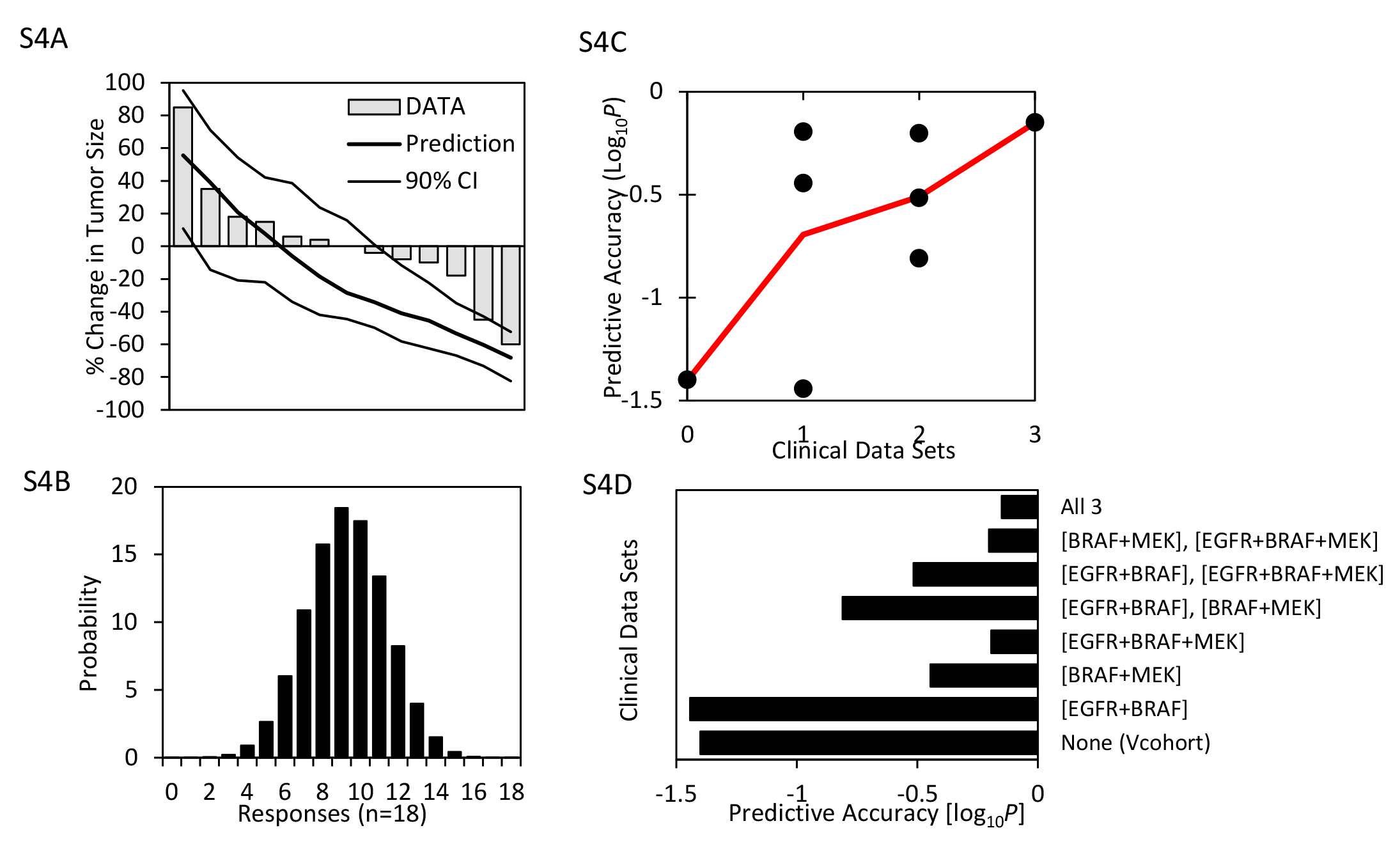

Supplement: Supplementary file 4 — Figure S4 [file 41540_2017_16_MOESM4_ESM.tif]

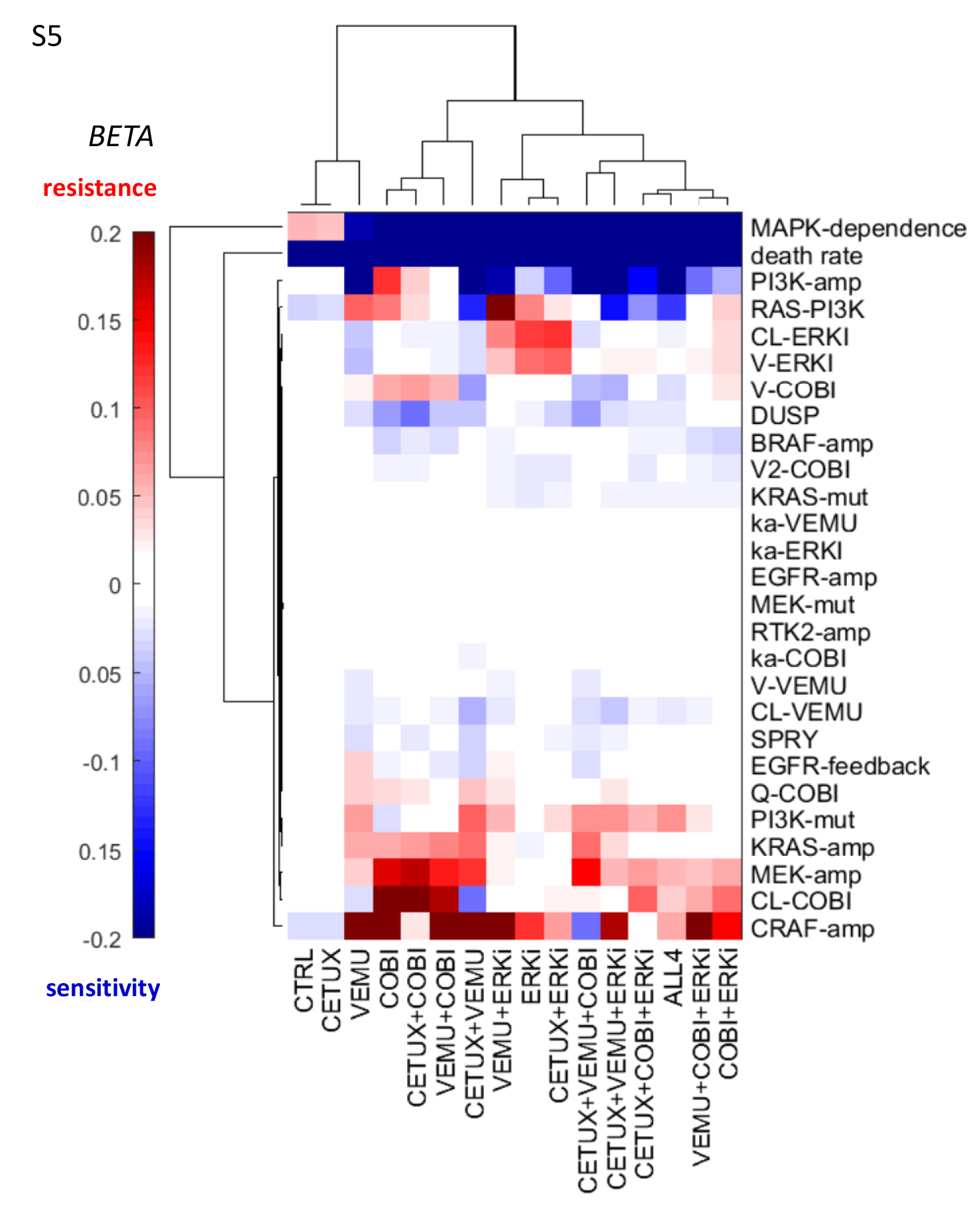

Supplement: Supplementary file 5 — Figure S5 [file 41540_2017_16_MOESM5_ESM.tif]
