# Supplementary material for: Clinical responses to ERK inhibition in BRAFV600E-mutant colorectal cancer predicted using a computational model
Source: NPJ Syst Biol Appl. 2017 Jun 2;3:14. doi: 10.1038/s41540-017-0016-1 (PMC5460205; doi:10.1038/s41540-017-0016-1)
Supplement: Supplementary file 11 — Supplementary Materials [file 41540_2017_16_MOESM11_ESM.docx]

**Supplementary Materials:**

Figure S1. Evaluation and exploration of in vitro model fitting results.

Fig S2. Expression (log_10_[RPKM]) of transcripts for 13 cell surface receptors across 11 *BRAF*^V600E^-mutant CRC cell lines, represented as a hierarchical clustered heatmap.

Figure S3. Simulated effects of individudal model parameter changes on HT29 tumor growth (A) and visual predictive checks (model simulations vs. data) for the HT29, CR1472, and CRC15 tumors (B-E).

Fig. S4. Predicted GDC-0994 responses based on the virtual cohort (lacking clinical data-based prevalence weights), and the prevalence-weighted virtual populations, generated using combinations of three clinical data sets.

Figure S5. Normalized multivariate regression coefficients (BETA) predictive of treatment sensitivity vs. resistance across the virtual tumor population.

Table S1. Model species

Table S2. Model parameters (in vitro)

Table S3. Digitized pMEK and pERK western blot data from Corocoran et al. ^32^

Table S4. Drug-target IC50 estimates

Table S5. Differentially expressed genes and pathway enrichment of cell lines

Table S6. Murine pharmacokinetic parameters

Table S7. In vivo (xenograft) model parameters

Table S8. Local Parameter Sensitivity Analysis based on the HT29 xenograft model

Table S9. Human population-pharmacokinetic parameters

Table S10. Virtual population parameters and Prevalence Weights

Table S11. Simulated ORRs to combination regimens and predicted synergy

VPop_Simulator.m. MATLAB script which loads and simulates the model to reproduce Fig 6.

MAPK_model.sbproj. Model structure encoded as a MATLAB SimBiology Project file

HillEQ.m. MATLAB function necessary to simulate the model

MAPK_model.xml. SBML version of the model. Note the original *.sbioproj* version repeatedly calls the external function (HillEQ.m) and requires the driver to map parameters and run simulations (VPop_Simulato.m), so this will not be functional, but will provide a structure for those wishing to implement the model using another open source platform.

**Supplementary Figure Legends:**

**Fig. S1.** Evaluation and exploration of in vitro model fitting results. (A) Simulated in vitro cell growth vs. pERK reslationship for 20 parameter sets (mean and std). (B,C) pMEK:pERK steady state relationships simulated with the Schoeberl 2002 model, while varying the range of forward (*f1*) vs. reverse (*r1*) reaction rates, and phosphatase (pAse) vs. pMEK concentrations, respectively.

**Fig S2.** Expression (log_10_[RPKM]) of transcripts for 13 cell surface receptors across 11 BRAF*^V600E^*-mutant CRC cell lines, represented as a hierarchical clustered heatmap.

**Fig. S3.** Simulated effects of changing individual model parameter values on HT29 tumor growth. (A) Sensitivity analysis on the HT29 model, with colors representing fold changes in 21-day tumor growth under the given treatment scenarios. Visual predictive checks (model simulations vs. data) for the HT29 (B), CR1472 alternative (Ci,ii) and CRC15 tumors (Ciii) xenografts.

**Fig. S4**. Predicted GDC-0994 responses based on the virtual cohort (lacking clinical data-based prevalence weights), and the prevalence-weighted virtual populations, generated using combinations of three clinical data sets. (A) Virtual cohort-based simulations of tumor size changes (waterfall plots) vs. data from 13 evaluable patients, and (B) distribution of expected responses for 18 patients (vs. 2/18 observed), are both inconsistent with observed responses to GDC-0994 monotherapy. (C, D) Accuracy of virtual population-based predictions of GDC-0994 monotherapy responses (rank-sum *P* values) using combinations of three available clinical data sets for the generation of prevalence weights.

**Fig. S5.** Normalized multivariate regression coefficients (*BETA*) predictive of treatment sensitivity vs. resistance across the virtual tumor population.
